# Polyene-Producing *Streptomyces* spp. From the Fungus-Growing Termite *Macrotermes barneyi* Exhibit High Inhibitory Activity Against the Antagonistic Fungus *Xylaria*

**DOI:** 10.3389/fmicb.2021.649962

**Published:** 2021-04-01

**Authors:** Jingjing Li, Moli Sang, Yutong Jiang, Jianhua Wei, Yulong Shen, Qihong Huang, Yaoyao Li, Jinfeng Ni

**Affiliations:** ^1^State Key Laboratory of Microbial Technology, Microbial Technology Institute, Shandong University, Qingdao, China; ^2^School of Pharmaceutical Sciences, Shandong University, Jinan, China

**Keywords:** fungus-growing termite, *Macrotermes barneyi*, *Streptomyces*, polyene, *Xylaria*

## Abstract

Fungus-growing termites are engaged in a tripartite mutualism with intestinal microbes and a monocultivar (*Termitomyces* sp.) in the fungus garden. The termites are often plagued by entomopathogen (*Metarhizium anisopliae*) and fungus garden is always threatened by competitors (*Xylaria* spp.). Here, we aim to understand the defensive role of intestinal microbes, the actinomycetes which were isolated from the gut of *Macrotermes barneyi*. We obtained 44 antifungal isolates, which showed moderate to strong inhibition to *Xylaria* sp. HPLC analysis indicated that different types of polyenes (tetraene, pentene, and heptaene) existed in the metabolites of 10 strong antifungal *Streptomyces* strains. Two pentene macrolides (pentamycin and 1′14-dihydroxyisochainin) were firstly purified from *Streptomyces* strain HF10, both exhibiting higher activity against *Xylaria* sp. and *M. anisopliae* than cultivar *Termitomyces*. Subsequently, tetraene and heptaene related gene disruption assay showed that the mutant strains lost the ability to produce corresponding polyenes, and they also had significantly decreased activities against *Xylaria* sp. and *M. anisopliae* compared to that of wild type strains. These results indicate that polyene-producing *Streptomyces* from the guts of *M. barneyi* have strong inhibition to competitor fungus and polyenes contribute to inhibitory effects on *Xylaria* sp.

## Introduction

Many fungus-growing insects (ants, termites, and southern pine beetles) actively cultivate one or two symbiotic fungi (Basidiomycota or Ascomycota) in an obligatory association (Chapela et al., 1994; Aanen et al., 2002; Hofstetter et al., 2006). The fungi serve as food for the insects, and the insects provide nutrients for fungus growth. However, the fungi cultivated by fungus-growing ants (Attini), fungus-growing termites (Macrotermitinae), and fungus-growing beetles (*Dendroctonus frontalis*) are plagued by specialized pathogens *Escovopsis* spp., *Xylaria* spp., and *Ophiostoma minus*, respectively (Currie et al., 1999a; Hofstetter et al., 2006; Ju and Hsieh, 2007). In addition, entomopathogenic fungi, such as *Beauveria bassiana* and *Metarhizium anisopliae*, are abundant in soil, and can be infectious to fungus-growing insects (Strasser et al., 2000; Mburu et al., 2009).

The multilateral symbioses systems of fungus-growing insects are compared to human agricultural systems. The symbiotic interactions in fungus-growing ant and beetle systems have been documented (Ramadhar et al., 2014). Microbial symbionts of fungus-growing ants and southern pine beetles, mainly *Pseudonocardia* and *Streptomyces* (Currie et al., 1999b; Cafaro et al., 2011), protect their fungal cultivars from competitor fungi via production of antifungal compounds, including dentigerumycin, gerumycins, antimycins, 9-methoxyrebeccamycin, cyphomycin, candicidin, mycangimycin, and macrolactam frontalamides (Scott et al., 2008; Haeder et al., 2009; Oh et al., 2009a, b; Blodgett et al., 2010; Schoenian et al., 2011; Sit et al., 2015; van Arnam et al., 2015; Chevrette et al., 2019b). Leaf-cutting ant-associated *Pseudonocardia* and *Amycolatopsis* isolates with antifungal activities protect hosts from entomopathogenic fungi (Sen et al., 2009; Mattoso et al., 2012).

Fungus-growing termites (Termitidae: Macrotermitinae), a group of higher termites, are abundant in tropical and subtropical regions of Asia and Africa (Liu et al., 2013). They have a significant effect on plant decomposition and element cycling (Li et al., 2017, 2021). The bacteria in termite mound soils could improve the fertility of the soil and suppress soil borne plant pathogens through the production of antibiotics and nutrient fixation, thus they might help reduce the farm use of chemical fertilizers and pesticides (Enagbonma and Babalola, 2019). Actinobacteria from nests of termite species, including *Macrotermes natalensis*, *Microtermes* sp., and *Odontotermes* sp., show higher antifungal activity against cultivar *Termitomyces* than *Xylaria* (Visser et al., 2012), while other Actinobacteria strains, or chemicals such as Actinomycin D and macrotermycins isolated from termite-associated actinomycetes exhibit selective antifungal activity against competitors (*Pseudoxylaria* or *Xylaria*) over *Termitomyces* (Beemelmanns et al., 2017; Yin et al., 2019). The role of actinobacteria in fungus-growing termites remains to be further explored.

*Macrotermes barneyi*, is a fungus-growing termite in the subfamily of Macrotermitinae, and widely distributed in southern China (Wu et al., 2012). *M. barneyi* lives in obligate symbiosis with a specialized fungal cultivar *Termitomyces* sp. (Basidiomycotina), which is the only visible fungus in active nests. *Xylaria* sp. thrives in abandoned termite nests and is the competitor of *Termitomyces* sp. Here, we assess the antifungal activity of actinomycetes through strain isolation, paired bioassay, HPLC analysis, compound identification, gene disruption and bioactivity assay. The results indicated that *M. barneyi* associated antifungal *Streptomyces* produced different types of polyenes, which contributes to the inhibitory activity against the antagonistic fungi.

## Materials and Methods

### Sample Collection and Tested Fungi

The workers and soldiers of *M. barneyi* were collected in July 2017 and June 2018 from termite nests in Hunan (E 112° 96′, N 26° 58′) and Guangdong (E 113° 60′, N 24° 82′), China. The termites were transferred into sterilized tubes and stored on dry ice. Gut dissection was performed within 48 h. The insect-pathogenic fungi *M. anisopliae* ACCC 30103 and *B. bassiana* ACCC 30730 were purchased from the Agricultural Culture Collection of China, Beijing, China. *Termitomyces* sp. and *Xylaria* sp. were isolated from the fungus gardens of *M. barneyi*. Strain identity was verified by PCR amplification and sequencing of the ITS gene using the primers ITS1 and ITS4 (Heine et al., 2018).

### Gut Dissection and DNA Extraction

Termite workers were surface sterilized by successive soaking in sterile water and phosphate buffered saline, and then rinsed with 70% ethanol and sterile water (Ramadhar et al., 2014). 150 workers and 50 soldiers were dissected. The workers guts were aseptically removed with forceps and divided into foregut, midgut, and hindgut, which were immersed in PBS buffer (NaCl 8 g/L, KCl 0.2 g/L, Na_2_HPO_4_ 1.42 g/L, KH_2_PO_4_ 0.27 g/L, pH 7.4) (Schmitt-Wagner et al., 2003). The samples were homogenized and transferred into sterile tubes to be used for actinomycetes isolation.

### Strain Isolation and Phylogenetic Analysis

Actinomycetes were isolated by the serial dilution method on Gause’s No.1, M2 (Mincer et al., 2002), M4 (Mincer et al., 2002), PY-CMC (Min et al., 1994), HVA (Subramani and Aalbersberg, 2013), and Chitin media (Benndorf et al., 2018). Fifty workers and soldiers were dissected to obtain gut samples. After shaking at 150 rpm for 30 min, gut suspensions of 10^–1^ to 10^–3^-fold dilutions were plated onto six isolation media with or without inhibitors (50 μg/mL potassium dichromate, 50 μg/mL cycloheximide, and 20 μg/mL nalidixic acid) (Malviya et al., 2014). The plates were incubated at 30°C for 7–15 days. Colonies with distinct morphological characteristics were transferred and purified on yeast extract-malt extract (ISP2) agar plates (Shirling and Gottlieb, 1966). Isolates were kept in 20% glycerol at −80°C for long-term preservation.

For genomic DNA extraction, actinomycetes were grown in nutrient-rich liquid Tryptic Soy Broth (TSB) medium at 30°C for 4 days. Cells were then harvested, and the genomic DNA was extracted using a bacterial DNA Extraction Kit (Omega, BioTek, United States). The 16S rRNA gene was amplified by PCR using general primers 27F and 1492R (Liu et al., 2013). Amplification reactions were standardized in a total volume of 50 μl containing 2× EasyTaq master mix (Takara, Dalian, China), 100 ng genomic DNA of isolated actinomycetes, 10 μM of each primer and sterile water. Cycle parameters for PCR were 10 min at 95°C (initial denaturation); 35 cycles at 95°C for 30 s, 58°C for 30 s and 72°C for 2 min; 7 min at 72°C (final elongation). The amplified 16S rRNA fragments were cloned into the pMD19-T vector. After sequencing, these complete 16S rRNA gene sequences were compared with available sequences in the GenBank database using the BLAST program in NCBI. The phylogenetic tree was constructed via the neighbor-joining tree algorithm using MEGA version 7.0 (Kumar et al., 2016). The confidence values of nodes in the trees were evaluated by 1000 bootstrap replicates (Felsenstein, 1985).

### Actinobacteria–Fungi Paired Challenge Assays

Actinobacteria-fungi paired bioassays were performed according to a previously reported method (Benndorf et al., 2018). Strains were grown at 30°C for 3 days in ISP2 or TSB. Aliquots of 20 μl liquid culture were used to inoculate on the centers of Potato Dextrose Agar (PDA) plates. Then, plates were inoculated at the edges with two agar pieces covered with fungal mycelium (*Xylaria* sp.). All assays were performed in triplicate. Plates were incubated for 7–10 days at 30°C and checked daily until a clear and stable zone of inhibition (ZOI) appeared (normally after 7 days). The ZOI values were given by measuring the distance of two inoculated *Xylaria*. The final ZOI is the average value of three replicates. The categories of strong inhibition (ZOI > 2 cm), moderate inhibition (ZOI 0.5–2 cm), and little or no inhibition (ZOI < 0.5 cm) were defined (Scott et al., 2008).

### Antifungal Assay of Crude Extracts

Actinomycetes isolates with strong antifungal activity against *Xylaria* sp. in Actinobacteria-fungi paired bioassays were cultivated in 40 ml TSB, MS (2% D mannitol, 2% soybean meal, pH 7.2) and ISP2 liquid media for 4 days at 30°C with shaking at 150 rpm. The liquid broth of 21 actinomycetes isolates was centrifuged at 12000 × *g* for 10 min. Culture supernatants were dried in vacuum rotary evaporator at 38°C and dissolved in 2 ml methanol, which was used as crude extracts. The assay was conducted using the agar diffusion method (Haeder et al., 2009). First, sterilized stainless-steel Oxford cups (10 × 6 × 8 mm) were placed on PDA plates. Next, PDA agar medium with fungal spores and mycelium was introduced into the upper plates. After solidification, the Oxford cups were removed, the crude extracts (100 μl) were added into each well. The diameters of the inhibition zones around the wells were observed after incubation for 1 days at 30°C. The active crude extracts were subjected to HPLC analysis.

### HPLC Analysis of the Active Crude Extracts

The active crude extracts were applied to HPLC (DIONEX Ultimate 3000 instrument) analysis. HPLC was operated at a flow rate of 1 mL/min with a C18 column (YMC-Pack Pro, 250 × 4.6 mm, 5 μm). Water-0.1% formic acid (solvent A) and acetonitrile-0.1% formic acid (solvent B) were used as the mobile phases. The column was eluted for a conventional separation by using elution gradient of 20% B, increased to 35% B in 3 min, 45% B in 10 min, 90% B in 20 min, and 100% B in 22 min (held for 3 min). Afterward, the elution gradient was reduced to 20% B in 27 min and sustained for 3 min.

### Genome Sequencing and Analysis

The *Streptomyces* strains were cultivated in 20 ml TSB liquid medium for 2 days at 30°C with shaking at 150 rpm. The culture broth (20 ml) was centrifuged at 12000 × *g* for 10 min, culture supernatant was removed and cell pellets were harvested. Genomic DNA was isolated from cell pellets that were physically ground in liquid N_2_ and then extracted using a bacterial DNA Extraction Kit (Omega, BioTek, United States). Whole-genome sequencing was performed using PacBio SMRT sequencing technology by the Novogene sequencing company. Biosynthetic gene clusters (BGCs) of secondary metabolites were predicted by antiSMASH 4.0 (Blin et al., 2017). The annotation of the polyene BGCs was performed using Blastp (non-redundant proteins).

### Isolation and Identification of the Active Compounds From *Streptomyces* sp. HF10

To isolate the antifungal compounds from *Streptomyces* sp. HF10, we set out a large-scale culture (20 L ISP7 broth) (Shirling and Gottlieb, 1966) for 4 days at 30°C with shaking at 160 rpm. The culture was centrifuged to obtain the supernatant followed by absorption using macroporous resin D101 overnight. The resin was loaded on a column and washed with water and then eluted with 50% methanol and 100% methanol, respectively. The antifungal activity of each fraction was tested. The active fractions were dried by evaporation to obtain the crude extract.

The crude extract was loaded on a middle-pressure liquid chromatography column (MPLC; 80 g RP-18 silica gel; 20, 40, 60, and 100% acetonitrile containing 0.1% formic acid, 200 ml for each gradient) to yield Fr.1-4. Fr 2 was subjected to Sephadex LH-20 to yield Fr.2a and 2b. Fr.2a and Fr.2b were subjected to MPLC (25%, 30% acetonitrile containing 0.1% formic acid) to yield Fr.2a1 and Fr.2b2. Fr.2a1 and Fr.2b2 were purified by semi-preparative reverse-phase HPLC (DIONEX Ultimate 3000 instrument, YMC-Pack Pro C18, 10 × 250 mm, 5 μm, flow rate 4 mL/min, UV detection at 350 nm) to yield 1 (10 mg, 38% acetonitrile containing 0.1% formic acid, *t*_*R*_ = 9.5 min) and 2 (9 mg, 29% acetonitrile containing 0.1% formic acid, *t*_*R*_ = 8.5 min), respectively. Subsequently, compounds 1 and 2 dissolved in methanol were subjected to high resolution mass spectrometry (HRMS) for determination of molecular mass. Compounds 1 and 2 were dissolved in DMSO-*d* to measure ^1^H, ^13^C-NMR and two-dimensional NMR spectra on a 600 MHz spectrometer.

### *In vitro* Antifungal Assay and Determination of Minimal Inhibitory Concentrations

Antifungal activity of the purified compounds was monitored by the paper disc diffusion method (Um et al., 2013). An agar piece covered with fungal mycelium (*Xylaria* sp. or *Termitomyces* sp.) was inoculated at the center of the PDA plates. Plates were incubated for 7 days at 30°C until a clear colony was apparent. Sterile discs were placed around the colony covered with different amounts (2.5, 5, and 10 μg) of purified pentamycin or 1′14-dihydroxyisochainin. DMSO was used as a negative control. All of the tested discs were observed on a daily basis for 3 days. A liquid antifungal assay was performed to measure minimal inhibitory concentrations (MICs) of compounds. To prepare mycelia or spore suspensions, *Xylaria* sp., *Termitomyces* sp. and two entomopathogens were cultivated on PDA plates 5–7 days, then mycelia or spores were collected and homogenized in potato dextrose broth medium to maintain the absorbance of OD_600_ approximately 0.5. The suspension was transferred into the wells of 96-well microplate followed by adding 2-fold serial dilution of purified compounds or amphotericin with a final concentration of 128, 64, 32, 16, 8, 4, 2, and 1 μg/mL. Subsequently, in order to determine the exact difference in antifungal activity of purified compounds against tested fungi, different concentrations of pentamycin (30, 25, and 20 μg/mL), 1′14-dihydroxyisochainin (150 145, and 140 μg/mL) or amphotericin (60, 55, 50, 45, and 40 μg/mL) were applied against *Termitomyces* sp. The above suspension without adding compound used as negative control was simultaneously cultured. The plates were incubated at 30°C and monitored for inhibition. The MIC value was calculated as the lowest concentration showing complete inhibition of the tested strain. All of the assays were performed in triplicate.

### Construction of Natamycin Biosynthetic Gene Disruption Mutant of *Streptomyces* sp. GS7

To inactivate the BGC of natamycin (*pim*) in *Streptomyces* sp. GS7, the polyketide synthase gene (*pimS0*) was replaced with a gene disruption cassette by homologous recombination. The gene disruption cassette containing a selectable apramycin resistance gene *aac(3)IV* and an origin of transfer gene *oriT* were jointly amplified by PCR from plasmid PIJ773 (Gust et al., 2003) with the primers (*aac(3)IV*-P4F/*aac(3)IV*-P4R). Two homologous arms flanking *pim*S0 were amplified from the genomic DNA of *Streptomyces* sp. GS7 with the primers (dP4LF/dP4LR, dP4RF/dP4RR). The above three fragments were assembled into pUC19 by Gibson assembly (Gibson et al., 2009), to yield pUC19-Δ*pim*S0. The constructed plasmid pUC19-Δ*pim*S0 was transformed into *Streptomyces* sp. GS7 by conjugation and the exconjugants were selected on MS agar medium with apramycin (Sangon Biotech, Shanghai, China). After three rounds of non-selective growth, the desired double cross-over mutants were confirmed by PCR. Amplification reactions were performed in a final volume of 25 μl containing 2× Rapid Taq master mix (Vazyme Biotech Co., Nanjing, China), 50 ng genomic DNA of mutants or wide-type strains, 10 μM of each primer (S0vF/S0vR), 5 μl PCR enhancer (Vazyme Biotech Co., Nanjing, China) and sterile water. PCR amplification program involved an initial DNA denaturation at 95°C for 10 min, followed by 35 cycles of denaturation at 95°C for 30 s, annealing at 58°C for 30 s and elongation at 72°C for 3 min 30s, which followed by a final extension at 72°C for 7 min. All primers were listed in Supplementary Table 7.

### Construction of the Candicidin Biosynthetic Gene Disruption Mutant of *Streptomyces* sp. GF20

To inactivate the BGC of candicidin (*fsc*) in *Streptomyces* sp. GF20, the polyketide synthase gene (*fsc*A) was replaced with a gene disruption cassette by homologous recombination. The gene disruption cassette *oriT-aac(3)IV* was amplified by PCR from plasmid pSET152 (Bierman et al., 1992) with the primers (*aac(3)IV-Xba*I-F/*aac(3)IV-Xba*I-F) and digested with *Xba*I. Two homologous arms flanking *fsc*A were amplified from the genomic DNA of *Streptomyces* sp. GF20 with the primers (dP7-*Pst*I-LF/dP7-*Xba*I-LR, dP7-*Xba*I-RF/dP7-*Hin*dIII-RR). Homologous arms were digested with *Pst*I/*Xba*I or *Hin*dIII/*Xba*I, respectively. The three fragments were ligated into pSPRam, which is pOJ260 (Bierman et al., 1992) derivative containing the reporter melanin gene *mel* (Wang et al., 2018) and resistance marker *aadA* (spectinomycin-resistance), to yield pSPRam-Δ*fsc*A. The constructed plasmid pSPRam-Δ*fsc*A was transformed into *Streptomyces* sp. GF20 by conjugation and exconjugants were selected on MS agar medium with apramycin. Then the cells were cultured on MS medium plates containing 5 mg/L copper sulfate and 100 mg/L tyrosine, white colonies as double cross-over mutants were picked (Wang et al., 2018). The double cross-over mutants were confirmed by PCR. The reaction systems were same as described early. Program parameters for PCR were 10 min at 98°C (initial denaturation); 35 cycles at 95°C for 30 s, 58°C for 30 s and 72°C for 6 min; 7 min at 72°C (final elongation). All used primers were listed in Supplementary Table 7.

## Results

### Isolation and Phylogenetic Analysis of Actinomycetes Strains

**FIGURE 1:**
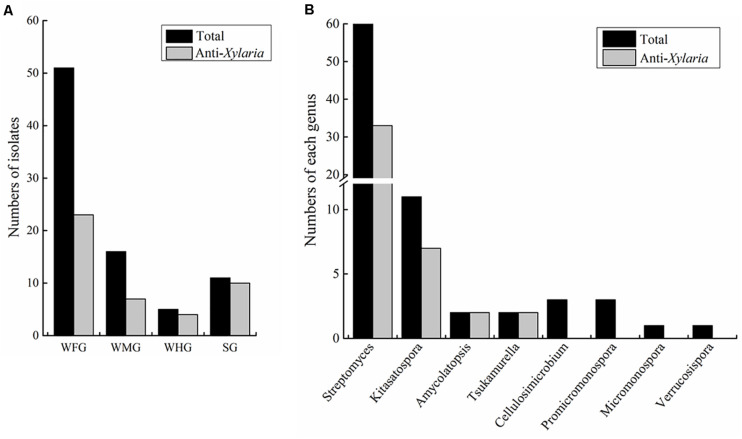
Statistics of Actinobacteria isolated from intestines of *Macrotermes barneyi*. **(A)** Total numbers of Actinobacteria isolates and those with anti-*Xylaria* activity from the worker foregut (WFG), worker midgut (WMG), worker hindgut (WHG), and the soldier gut (SG), respectively. **(B)** Numbers of Actinobacteria isolates and those with anti-*Xylaria* activity in each genus. The anti-*Xylaria* activity was examined by the paired challenge assay.

**FIGURE 2:**
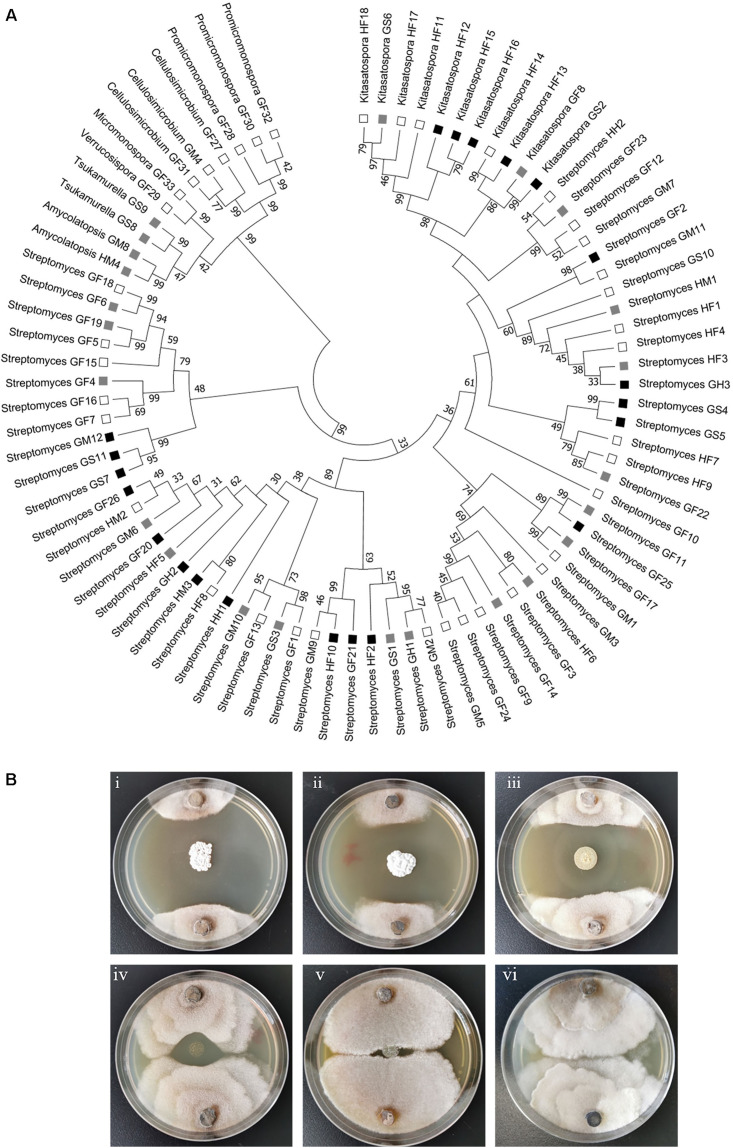
Phylogenetic and anti-*Xylaria* activity analyses of the isolated actinomycetes. **(A)** Phylogenetic tree of all the isolated strains. The tree was constructed with Mega 7.0 by the Neighbor-Joining method. The branch values indicate bootstrap support (>30 are given) of 1000 replicates. The ability to inhibit the growth of *Xylaria* sp. is indicated with black squares (strong inhibition, ZOI > 2 cm), gray squares (moderate inhibition, ZOI = 0.5–2 cm), and open squares (little or no inhibition, ZOI < 0.5 cm) (Scott et al., 2008). HF: Hunan foregut, HM: Hunan midgut, HH: Hunan hindgut, GF: Guangdong foregut, GM: Guangdong midgut, GH: Guangdong hindgut, GS: Guangdong soldier. **(B)** Examination of the anti-*Xylaria* activity of the isolated actinomycetes by paired challenge assay. Inhibition of representative *Streptomyces* against *Xylaria* sp. is shown. (i–v) *Streptomyces* sp. strains against *Xylaria* sp. (i–iii) HF10, GS7, and GF20, strong inhibition; (iv) GM6, moderate inhibition; (v) GM1, no inhibition; (vi) control, *Xylaria* sp.

Actinobacteria strains were isolated from the foregut, midgut and hindgut of workers as well as the gut of soldiers. According to distinct morphological features of actinomycetes and 16S rRNA gene sequence analysis, in total, we obtained 83 strains of actinomycetes belonging to eight genera from two termite samples collected from Hunan and Guangdong (Figure 1 and Supplementary Table 1). Among these, 72 strains were isolated from workers, and 11 from soldiers. Overall, 60 *Streptomyces* strains were isolated from the foregut, midgut and hindgut of workers and the gut of soldiers. 11 *Kitasatospora* strains were isolated from the foregut of workers and the gut of soldiers (Figure 1). Defined by a threshold of <98.65% sequence similarity, seven putative new Actinobacteria species (*Streptomyces*, *Kitasatospora*, and *Amycolatopsis*) were obtained from termite workers (Supplementary Table 2). Among them, six strains (HF5, HF17, GF5, GF6, GF15, and GF18) were isolated from the foregut and one strain GM8 from the midgut of *M. barneyi*. Phylogenetic analysis of all of the isolates based on 16S rRNA sequences revealed that the isolates formed two clades within the Actinobacteria phylum (Figure 2A), *Streptomyces* and *Kitasatospora* isolates clustering into one clade and the remaining 12 isolates clustering into another clade.

### The Isolated Actinomycetes Exhibited Antifungal Activity Against *Xylaria* sp.

To explore the antifungal activity of the isolated strains, all the Actinobacteria isolates were challenged against fungal garden competitor *Xylaria* sp. Forty-four actinomycetes isolates, which belongs to four genera (*Streptomyces*, *Kitasatospora*, *Amycolatopsis*, and *Tsukamurella*), exhibited moderate to strong antifungal activity against *Xylaria* sp. (Figure 2A). Among these, 23, 7, and 4 strains were isolated from foregut, midgut, and hindgut of workers, respectively, and 10 strains were isolated from the gut of soldiers. Twenty-one isolates with strong antifungal activity belong to two genera (*Streptomyces* and *Kitasatospora*) and cluster into one clade (Figure 2A). It was noted that *Streptomyces* strains GF2 and GM11 have the same closest type strain (*Streptomyces drozdowiczii* NRRL B-24297), HF10 and GM9 have the same type strain (*Streptomyces misionensis* JCM 4497), GF26 and HM2 have the same type strain (*Streptomyces sampsonii* ATCC 25495) in the blast search in NCBI (Supplementary Table 1), GF2, HF10, and GF26 presented antifungal activity while GM11, GM9, and HM2 had no antifungal activity (Figure 2A), which was supported by the study that taxonomic and metabolic incongruence exists in *Streptomyces* (Chevrette et al., 2019a).

Paired challenge assays of five representative strains were shown in Figure 2B. As shown in this figure, three *Streptomyces* isolates (HF10, GS7, and GF20) exhibited strong inhibition against *Xylaria* sp. (Figure 2Bi–iii). *Streptomyces* sp. GM6 exhibited moderate inhibition against *Xylaria* sp. and *Streptomyces* sp. GM1 displayed no inhibition against *Xylaria* sp. (Figure 2Biv,v).

### Polyene Compounds Were Detected in the Metabolites of Majority of the *Streptomyces* Strains

To identify putative compounds responsible for the antifungal activity, 21 actinomycetes strains with strong antifungal activity against *Xylaria* sp. were selected and cultivated in TSB, MS, and ISP2 liquid media, respectively. Agar diffusion assay showed that the fermentation broth of 15 strains had antifungal activity against *M. anisopliae*. Here *M. anisopliae* was used instead of *Xylaria* sp. owing to the instability of crude extracts. The metabolites of these strains were analyzed by HPLC and the profiles of 10 out of the 15 *Streptomyces* strains, GS7, GS11, GM12, HF10, GF21, GF25, HM3, GF20, GF26, and GH2, showed peaks with typical UV/vis spectra of four type of polyenes (Table 1 and Supplementary Figures 1–10). Among these strains, GS7, GS11, and GM12 showed tetraene peaks (292, 305, and 320 nm) (Supplementary Figures 1–3; Mendes et al., 2001); HF10, GF21, and GF25 showed pentene peaks (325, 340, and 358 nm) (Supplementary Figures 4–6; Xiong et al., 2012); HM-3 showed linear heptaene peaks (354, 272, and 393 nm) (Supplementary Figure 7; Oh et al., 2009b); GF20, GF26, and GH2 showed heptaene peaks (360, 381, and 406 nm) (Supplementary Figures 8–10; Haeder et al., 2009). While no obvious polyene peaks were observed in the metabolite profiles of the remaining five strains (*Kitasatospora* sp. HF13, HF15, and GS2, *Streptomyces* sp. HH1 and GS5).

**TABLE 1:** Predicted polyenes from antifungal crude extracts of isolated actinomycetes.

Strains	Isolated resource	Antifungal activity^*a*^	Absorption peaks (nm)	Polyene type	References
*Streptomyces* GS7	Solider gut	+++	293,306,320	Cyclic tetraene	Mendes et al., 2001
*Streptomyces* GS11	Solider gut	+++	293,306,320	Cyclic tetraene	Mendes et al., 2001
*Streptomyces* GM12	Worker midgut	+++	294,306,321	Cyclic tetraene	Mendes et al., 2001
*Streptomyces* HF10	Worker foregut	+++	324,340,358	Cyclic pentene	Xiong et al., 2012
*Streptomyces* GF21	Worker foregut	++	325,340,358	Cyclic pentene	Xiong et al., 2012
*Streptomyces* GF25	Worker foregut	++	325,340,358	Cyclic pentene	Xiong et al., 2012
*Streptomyces* HM3	Worker midgut	+++	353,372,393	Linear heptaene	Oh et al., 2009b
*Streptomyces* GF20	Worker foregut	+++	360,381,400	Cyclic heptaene	Haeder et al., 2009
*Streptomyces* GF26	Worker foregut	++	360,382,401	Cyclic heptaene	Haeder et al., 2009
*Streptomyces* GH2	Worker hindgut	+	360,382,401	Cyclic heptaene	Haeder et al., 2009
*Kitasatospora* HF13	Worker foregut	+++	–	–	–
*Kitasatospora* HF15	Worker foregut	+++	–	–	–
*Streptomyces* HH1	Worker hindgut	++	–	–	–
*Kitasatospora* GS2	Solider gut	++	–	–	–
*Streptomyces* GS5	Solider gut	+	–	–	–

*^a^The antifungal activity against Metarhizium anisopliae. IZD: Inhibition zone diameter excluding width of Oxford cups. +++: IZD ≥ 1.5 cm. ++: IZD ≥ 1 cm. +: IZD ≥ 0.5 cm.*

### Prediction of BGC and Purification of Antifungal Polyene Compounds From *Streptomyces* sp. HF10 and Bioactivity Assays of Purified Compounds

**FIGURE 3:**
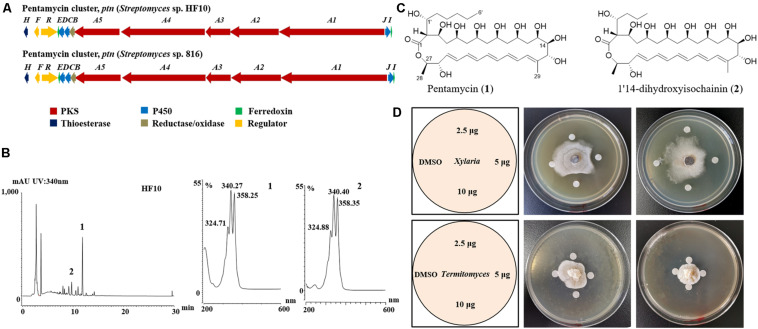
*Streptomyces* sp. strain HF10 produces antibiotics pentamycin and 1′14-dihydroxyisochainin. **(A)** The genetic organization of the pentamycin BGC of strain HF10 compared with the reported one in *Streptomyces* sp. 816. The BGC of pentamycin in strain HF10 was identified by whole genome sequencing and bioinformatic analysis. **(B)** HPLC profiles of metabolites from strain HF10 (left) and UV/vis spectra of compounds 1 and 2 (right). **(C)** The chemical structures of pentamycin (1) and 1′14-dihydroxyisochainin. (2). **(D)** Antifungal activity of pentamycin and 1′14-dihydroxyisochainin by disc diffusion assay. Pentamycin and 1′14-dihydroxyisochainin were purified from strain HF10 as described in section “Materials and Methods.” Pentamycin (middle) and 1′14-dihydroxyisochainin (right) against *Xylaria* sp. Pentamycin (middle) and 1′14-dihydroxyisochainin (right) against *Termitomyces* sp. Left, schematic map showing sample arrangement in experiments. A gradient amount (2.5, 5.0, and 10 μg) of pentamycin or 1′14-dihydroxyisochainin was used. An agar piece covered with fungal mycelium (*Xylaria* sp. or *Termitomyces* sp.) was inoculated at the center of the PDA plate for 7 days at 30°C. Sterile discs containing the compounds dissolved in DMSO were placed around the colony. The plates were cultured at 30°C for 3 days and observed on daily basis.

To identify polyene BGCs, whole genome sequencing of strain HF10 was performed. Comparative analysis revealed that the genetic organization of the target gene cluster in strain HF10 was almost identical to the reported pentamycin gene cluster in *Streptomyces* sp. S816 (Figure 3A and Supplementary Table 3; Zhou et al., 2019).

To identify the antifungal compounds produced by strain HF10 which had the strongest activity against *Xylaria* sp. as observed in the paired bioassay (Figure 2B), we performed a scale-up fermentation of strain HF10 and obtained 10 mg of pure compound 1 and 9 mg of pure compound 2 after a series of column chromatography purifications (Figure 3B). The relative molecular masses of compounds 1 and 2 were detected at m/z 670.3936 (1) and m/z 642.3630 (2) by HRMS, respectively (Supplementary Figures 11, 12). The ^1^H- and ^13^C-NMR data of 1 and 2 were summarized in Supplementary Table 4 (Supplementary Figures 13–20; Li et al., 1989). Based on these results, the chemical structures of 1 and 2 were determined to be pentamycin and 1′14-dihydroxyisochainin, respectively (Figure 3C).

In paper disc diffusion assays, pentamycin (1) exhibited stronger activity against *Xylaria* sp. (Figure 3D middle) than *Termitomyces* sp. (Figure 3D middle). With the tested concentrations, 1′14-dihydroxyisochainin (2) also selectively suppressed *Xylaria* sp. (Figure 3D right) rather than *Termitomyces* sp. (Figure 3D right). In the MIC determination assays, the pentamycin (1) exhibited the lowest MIC (4 μg/mL) against *Xylaria* sp., while the MIC of amphotericin B against *Xylaria* sp. was 16 μg/mL (Table 2). The pentamycin consistently inhibited the growth of *Xylaria* sp., *M. anisopliae*, and *B. bassiana* more strongly than that of *Termitomyces* sp. While, 1′14-dihydroxyisochainin selectively suppressed the growth of pathogens but not *Termitomyces* sp. Thus, the antifungal activities of the two polyene compounds against *Xylaria* sp. and entomopathogens were stronger than that of the cultivar.

**TABLE 2:** Minimal inhibitory concentrations (MIC, μg/mL) of pentamycin and 1′14-dihydroxyisochainin.

Strains	MIC (μg/mL)		
	Pentamycin	1′14-dihydroxyisochainin	Amphotericin B
*M. anisopliae* ACCC 30103	8	64	16
*B. bassiana* ACCC 30730	8	64	32
*Xylaria* sp.	4	32	16
*Termitomyces* sp.	20	>150	60

*Amphotericin B was used as a positive control.*

### Mutants Completely Abolished the Polyene Production and the Activities of the Mutants Against Antagonistic Fungi Were Greatly Weakened

**FIGURE 4:**
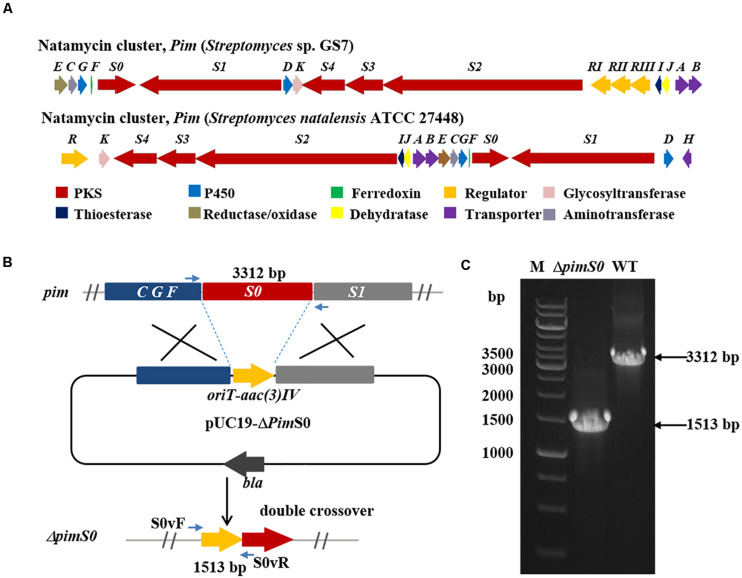
Disruption of a key natamycin biosynthetic gene *pimS0* in *Streptomyces* sp. GS7. **(A)** Comparison of the genetic organization of natamycin BGCs in *Streptomyces* sp. GS7 and *S. natalensis* ATCC 27448. **(B)** Schematic representation for disruption of *pimS0*. **(C)** Verification of the *pimS0*-disrupted mutant by PCR. M, DNA marker. The fragments were amplified using primers of S0vF/S0vR.

**FIGURE 5:**
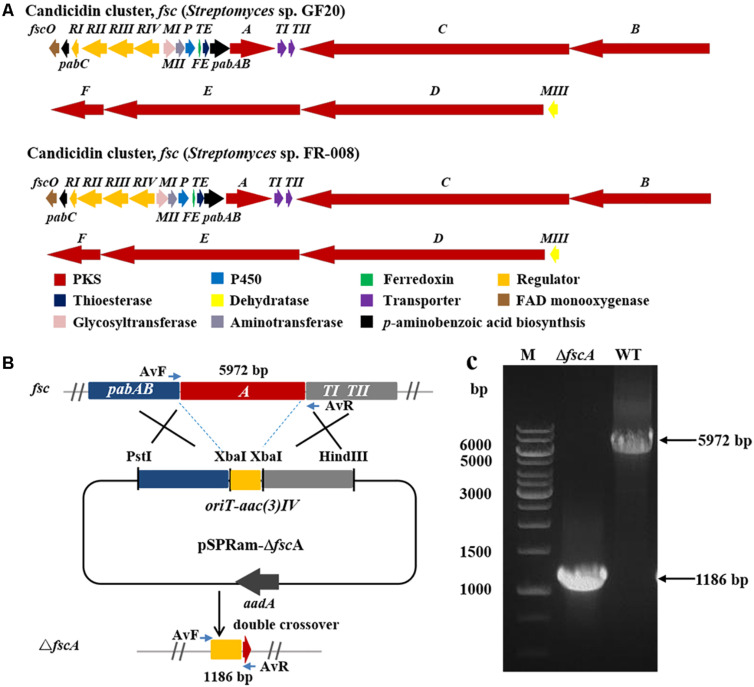
Disruption of candicidin biosynthetic gene *fscA* in *Streptomyces* sp. GF20. **(A)** Comparison of the genetic organization of candicidin BGCs in *Streptomyces* sp. GF20 and *Streptomyces* sp. FR-008. **(B)** Schematic representation for disruption of *fscA* in *Streptomyces* sp. GF20. **(C)** Verification of the *fscA*-disrupted mutant by PCR. M, DNA size marker. The fragments were amplified using primers of AvF/AvR.

Two *Streptomyces* strains, GS7 and GF20 (Figure 2B), producing tetraene and heptaene compounds (Supplementary Figures 1, 8) revealed by HPLC analysis, were subjected to genome sequencing. The sequence alignment showed that the putative tetraene cluster in GS7 was homologous to the natamycin synthesis cluster of *Streptomyces natalensis* ATCC 27448 and showed 77% sequence identity (Figure 4A; Mendes et al., 2001). The putative heptaene cluster in GF20 was homologous to the candicidin synthesis cluster of *Streptomyces* sp. FR-008 and showed 100% sequence identity (Figure 5A; Chen et al., 2003). Potential ORFs responsible for natamycin and candicidin biosynthesis were shown in Supplementary Tables 5,6.

**FIGURE 6:**
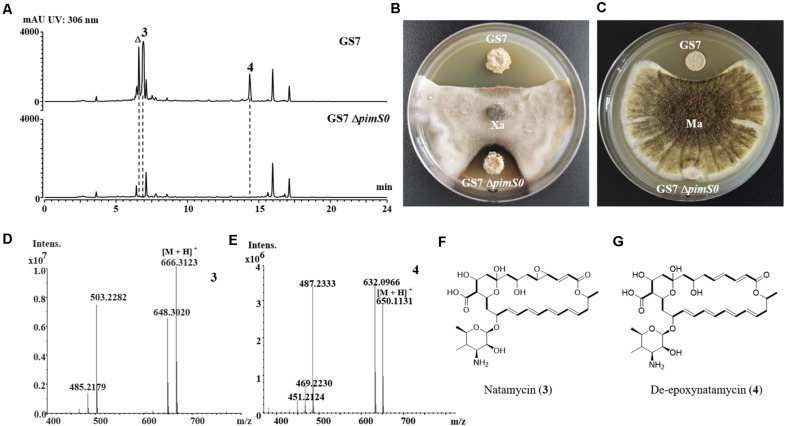
The deletion mutant of natamycins in *Streptomyces* sp. GS7 had weaker antifungal activities against *Xylaria* sp. and *Metarhizium anisopliae*. **(A)** HPLC profiles of the metabolites of *Streptomyces* sp. GS7 wild type and the GS7Δ*pimS0* mutant. The triangle symbol (Δ) denotes a homolog of natamycin. **(B,C)** Paired challenge assays of GS7 and the deletion mutant (GS7Δ*pimS0*) against *Xylaria* sp. (Xa, **B**) and *M. anisopliae* (Ma, **C**). **(D,E)** HRMS spectra of compounds 3 and 4 from GS7. **(F,G)** The predicted chemical structures of natamycin/pimaricin (3) and de-epoxynatamycin/de-epoxypimaricin (4).

**FIGURE 7:**
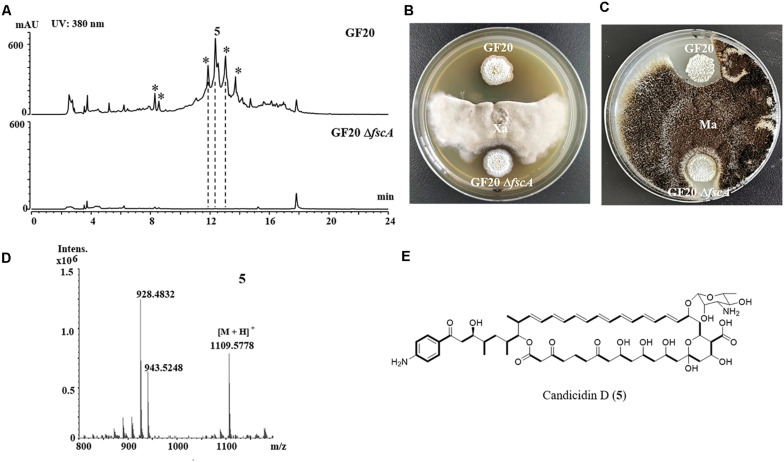
The deletion mutant of candicidins in *Streptomyces* sp. GF20 had weaker antifungal activities against *Xylaria* sp. and *M. anisopliae*. **(A)** HPLC profiles of the metabolites of wide type *Streptomyces* sp. GF20 and mutant GF20 Δ*fscA*. The asterisk symbols (*) denote homologs of candicidin. **(B,C)**, paired challenge assays of GF20 and the deletion mutant (GF20 Δ*fscA*) against *Xylaria* sp. (Xa, **B**) and *M. anisopliae* (Ma, **C**). **(D)** HRMS spectrum of compound 5 from GF20. **(E)** The predicted structure of candicidin D (5).

The 3.2 kb fragment of *pimS0* and 5.7 kb fragment of *fscA*, corresponding to the polyketide synthase gene of polyenes BGCs *pim* (natamycins) and *fsc* (candicidins) in strains GS7 and GF20, respectively, have been replaced with the gene disruption cassette (Figures 4B, 5B). Two mutants GS7Δ*pimS0* and GF20Δ*fsc*A generated by double cross-over were obtained and verified by PCR (Figures 4C, 5C) with primers S0vF/S0vR and AvF/AvR, respectively (Supplementary Table 7). HPLC analysis showed that two mutant strains were completely unable to produce compounds 3, 4 (Figure 6A), and 5 (Figure 7A). *Streptomyces*-fungi paired bioassays showed that both mutants (GS7Δ*pimS0* and GF20Δ*fsc*A) had significantly less inhibitory effects on the growth of *Xylaria* sp. and *M. anisopliae* than the corresponding wild type strains (Figures 6B,C, 7B,C). In addition, both wild type and mutant strains, exhibited stronger inhibition to *Xylaria* sp. than *M. anisopliae*. The results suggested that tetraene and heptaene, produced by *Streptomyces* sp. GS7 and GF20, respectively, are major antifungal compounds against the pathogens (*Xylaria* sp. and *M. anisopliae*), and compared to *M. anisopliae*, *Streptomyces* tends to inhibit *Xylaria* sp. more strongly than *M. anisopliae*.

The relative molecular masses of compounds 3, 4, and 5 were m/z 665.3123, 649.1131, and 1108.5778, respectively (Figures 6D,E, 7D). UV/Vis spectra of compound 3 and 4 were identical to pimaricin and de-epoxypimaricin from *S. natalensis* ATCC 27448, and compound 5 was identical to candicidin D from *Streptomyces* sp. FR-008 (Mendes et al., 2001; Chen et al., 2003). The identity of BGC, UV/Vis spectrum and relative molecular mass indicated that compounds 3, 4, and 5 are probably natamycin/pimaricin, de-epoxynatamycin/de-epoxypimaricin, and candicidin D, respectively (Figures 6F,G, 7E).

## Discussion

The success of termite fungiculture depends on multiple factors, such as the control of pathogens within termite fungus farms, termite antimicrobial peptides and gut bacteria with antimicrobial properties (Um et al., 2013; Poulsen, 2015; Bodawatta et al., 2019). The results in this study revealed that fungus-growing termite *M. barneyi*-associated actinomycetes produced different type of polyenes, which greatly inhibited competitor fungus *Xylaria* sp.

### Actinobacteria Isolation From the Digestive Tract of *M. barneyi*

The actinomycetes were isolated from *M. barneyi* workers and soldiers. Owing to relative smaller size of soldier guts than that of workers, also a fewer numbers of soldier collected than workers, only worker guts were separated into the different gut sections (foregut, midgut, and hindgut) (Figure 1A). By a culture-based approach, we obtained 83 Actinobacteria strains from the guts of *M. barneyi*, which belongs to 8 genera but mainly *Streptomyces* and *Kitasatospora* (Figure 1B). Previously, actinomycetes of 4 genera including *Streptomyces*, *Cellulosimicrobium*, *Promicromonospora*, and *Micromonospora* have been isolated from workers intestines of *M. natalensis* and *Odontotermes formosanus* (Benndorf et al., 2018), and genus *Kitasatospora* strains have been isolated from the cuticle of fungus-growing termite (Visser et al., 2012). To our knowledge, this is the first time to obtain actinomycetes isolates of three genera *Amycolatopsis*, *Tsukamurella*, and *Verrucosispora* from fungus-growing termites.

Based on 16S rRNA sequence analysis, seven strains were predicted to be potential novel actinomycetes (Supplementary Table 1). Recently, a number of new actinomycetes have been isolated and identified from the worker gut of *M. natalensis* (Benndorf et al., 2020a, b; Schwitalla et al., 2020). The intestinal microflora of fungus-growing termite represents a promising resource of novel actinomycetes.

It was noted there were relatively higher numbers of strains obtained from the foregut of worker than other gut sections. The possible explanation is: the variety and abundance of hindgut microflora are highest among three gut sections (Chew et al., 2018), and actinomycetes are at very low level in termite guts (Otani et al., 2014), thus on the plates spreading with hindgut sample, a large number of bacteria overgrew and slow-growing actinomycetes were difficult to grow. Therefore, there was few actinomycetes obtained from the hindgut. Pre-treatments of hindgut samples by drying heating (Bredholt et al., 2008) or adding inhibitor such as chloramine-T (Hayakawa et al., 1997) may facilitate the selective isolation of actinomycetes from the hindgut. Although majority of actinomycetes were isolated from the foregut, considering the foregut is the first gut section of ingested food, we could not preclude these actinomyces originate from the nest environment, where many actinobacteria strains exist (Visser et al., 2012).

### Antifungal Actinobacteria Strains From the Digestive Tract of *M. barneyi* Worker and Soldier

Plate paired assay revealed that 53% of the actinomycetes, mainly *Streptomyces* and *Kitasatospora* genus possessed antifungal activity against antagonistic fungus *Xylaria*. Majority of these antifungal actinomycetes were isolated from worker intestines, which could be explained by the fact that the workers have a greater chance of being exposed to pathogens than that of soldiers, since workers are responsible for transporting and digesting external plant material (Li et al., 2017). Additionally, the defensive microbes (most likely Actinobacteria and *Bacillus*) in the gut is helpful to selectively inhibit the potential antagonists when the substrate first passes through the worker gut and avoid the entry of pathogens into fungal comb (Um et al., 2013; Poulsen, 2015).

Soldiers mainly play a defensive role in the colony by using their large and strong mandibles as well as by chemical substances secreted from a frontal gland on the head (He et al., 2018). In the present study, we also obtained *Streptomyces* with antifungal activity against *Xylaria* sp. from soldier guts. Previous study of gut bacterial metagenomic analysis by Poulsen revealed that the soldiers have nearly similar or a bit higher relative abundance of *Streptomyces* than workers in *M. natalensis* (Poulsen et al., 2014). To our knowledge, this is the first time to obtain the *Streptomyces* isolates from the soldier, considering the relatively high percentage of antifungal strains, which suggest the potential role of soldier-associated *Streptomyces* in fungus comb against antagonistic fungus *Xylaria*.

### Polyenes-Producing *Streptomyces* Contribution to Inhibition of *Xylaria*

A key strategy of insects coping with environmental threats is the use of molecular defenses from symbiotic microbes (van Arnam et al., 2018), especially from insect-associated *Streptomyces* and *Pseudonocardia* (Scott et al., 2008; Haeder et al., 2009; Oh et al., 2009a, b; Blodgett et al., 2010; Schoenian et al., 2011; Sit et al., 2015; van Arnam et al., 2015; Chevrette et al., 2019b). To identify putative compounds responsible for the antifungal activity, the metabolites of antifungal strains were analyzed by HPLC, and the results revealed that the ten antifungal *Streptomyces* strains produce four type of polyenes with different number of conjugated double bonds. Several polyene compounds, including candicidin (Haeder et al., 2009; Barke et al., 2010), nystatin P1 (Barke et al., 2010), selvamicin (van Arnam et al., 2016), filipins (Gao et al., 2014), and mycangimycin (Oh et al., 2009b) have been reported from the symbiont actinomycetes of fungus-farming ants and southern pine beetles. *Bacillus* sp. from *M. natalensis* produced a polyene polyketide, bacillaene, which selectively inhibits antagonistic fungus of *Termitomyces* (Um et al., 2013), However, as far as we know, polyene compounds have not previously been isolated from fungus-growing termites-associated actinomycetes (Bi et al., 2011; Carr et al., 2012; Zhang et al., 2013, 2020; Kim et al., 2014; Benndorf et al., 2018; Klassen et al., 2019; Guo et al., 2020). Three strains with strong antifungal activities and potential polyene products corresponding to tetraene, pentene and heptaene, respectively, were subjected to bulk culture, probably due to chemical instability of polyenes (Worthen et al., 2001), finally only two pentene compounds (pentamycin and 1′14-dihydroxyisochainin) were purified from *Streptomyces* sp. HF10 (Figure 3C). Pentamycin (also called fungichromin) and 1′14-dihydroxyisochainin belong to polyene macrolides, containing the antifungal antibiotics amphotericin B (Sun et al., 2015) and nystatin (Fjaervik and Zotchev, 2005). Previously, fungichromin have been isolated from endophytic actinomycetes (Human et al., 2016) and lower termite-associated actinomycetes (Mevers et al., 2017). The compound 1′14-dihydroxyisochainin is an analog of chainin, which was first isolated from a soil actinomycete with antifungal activity against phytopathogens (Thirumalachar, 1955; Gopalkrishnan et al., 1968).

Although two purified pentene compounds are not novel products, they represent the first report on polyene compound from fungus-growing termite-associated *Streptomyces*. Bioactivity assays showed that the competitor fungus *Xylaria* sp. was the most susceptible to inhibition of two pentene compounds, compared with entomopathogen (*B. bassiana* and *M. anisopliae*) and fungal cultivar *Termitomyces* sp., which was similar to the studies in leaf-cutting ants and the southern pine beetle (Haeder et al., 2009; Oh et al., 2009b). Candicidin identified from ant-associated *Streptomyces* is highly active against pathogenic fungus *Escovopsis* sp. (Haeder et al., 2009). Our results suggest the potential role of the pentamycin in protecting the fungus comb of *M. barneyi* against competitor fungus *Xylaria* sp.

Since we failed to obtain tetraene and heptaene compounds from cultures of *Streptomyces* sp. GS7 and GF20, disruptive mutants of tetraene and heptaene BGCs were constructed (Figures 4, 5). HPLC analysis revealed that the corresponding polyene peaks disappeared and paired challenge assays showed the mutants had obviously weaker activities against antagonistic fungus *Xylaria* than the wild type strains, suggesting that tetraene and heptaene compounds produced by GS7 and GF20 contribute to the inhibition against the antagonistic fungi. Furthermore, both the wild type and mutant strains exhibited stronger inhibitory effects on antagonistic fungus (*Xylaria* sp.) than entomopathogen (*M. anisopliae*) (Figures 6B,C, 7B,C), suggesting tetraene and heptaene produced by *Streptomyces* firstly selectively inhibited the fungus comb antagonistic fungus *Xylaria*. Interestingly, the recent study by Bodawatta et al. (2019) showed that *M. natalensis* foraging workers significantly avoided the mycopathogen-exposed substrates, and did not show any preference between entomopathogen-exposed and control substrate. Overall, the present study by pentene compound purification, tetraene and heptaene BGCs gene disruption and bioactivity assays suggest that polyenes produced by *M. barneyi*-associated *Streptomyces* greatly contribute to inhibition of antagonistic fungi.

It was noted that mutants retained slight activity against *Xylaria* sp. after disruption of tetraene and heptaene BGCs (Figures 6B, 7B). Thus, except for polyenes, some other active compounds also inhibit the antagonistic fungi. In leaf-cutting ants, different antifungal secondary metabolites exhibited strong synergistic effects against pathogenic fungi (Schoenian et al., 2011). We inferred that in termite guts multiple compounds including polyenes and non-polyenes complement and reinforce the activities against pathogens.

The complex web of interactions involving insects (Poulsen et al., 2014), their fungal crops (Wang et al., 2015; Otani et al., 2019), specialized pathogens (Guo et al., 2016), symbiotic fungus (Xu et al., 2020), and symbiotic bacteria has become both a model system for chemical ecology and a source of naturally occurring small molecules. We are still a long way from identifying additional antifungal compounds in this system and understand thoroughly the chemical basis of symbiotic or antagonistic associations among termites, fungal cultivar, cultivar competitors, entomopathogenic fungi and antibiotic-producing actinomycetes.

## Conclusion

In conclusion, through actinomycetes isolation, bioactivity assays, active product purification, and BGCs gene disruption analysis, we show that *Streptomyces* isolated from the gut of fungus-growing termite *M. barneyi* are capable of producing a variety of polyenes, which significantly inhibit antagonistic fungus *Xylaria* over entomopathogenic fungi and fungal cultivar *Termitomyces*. The results indicate that potential role of different type of polyenes produced by *Streptomyces* in protection of fungus comb against the antagonistic fungus.

## Data Availability Statement

The complete 16S rRNA genes of 83 isolated actinomycetes in this study were deposited in GenBank (MN826234-MN826316). The GenBank accession numbers for the genomes of the three strains are CP047144-CP047145 (*Streptomyces* sp. HF10), CP047146 (*Streptomyces* sp. GS7), and CP047147 (*Streptomyces* sp. GF20).

## Author Contributions

JL, MS, YJ, and JW performed the experiments. JN, YL, and JL designed the experiments. JL and JN analyzed the data and wrote the manuscript. YL, YS, and QH helped to revision of the manuscript. All authors contributed to the article and approved the submitted version.

## Conflict of Interest

The authors declare that the research was conducted in the absence of any commercial or financial relationships that could be construed as a potential conflict of interest.
