# Supplementary material for: Polyene-Producing Streptomyces spp. From the Fungus-Growing Termite Macrotermes barneyi Exhibit High Inhibitory Activity Against the Antagonistic Fungus Xylaria
Source: Front Microbiol. 2021 Apr 1;12:649962. doi: 10.3389/fmicb.2021.649962 (PMC8047067; doi:10.3389/fmicb.2021.649962)
Supplement: Supplementary file 1 [file Data_Sheet_1.DOCX]

**Supplementary Information**

Polyene-producing *Streptomyces* spp. from the fungus-growing termite *Macrotermes barneyi* exhibit high inhibitory activity against the antagonistic fungus *Xylaria*

Jingjing Li^1^, Moli Sang^1^, Yutong Jiang^1^, Jianhua Wei^1^, Yulong Shen^1^, Qihong Huang^1^, Yaoyao Li^1, 2^*and Jinfeng Ni^1^*

^1^State Key Laboratory of Microbial Technology, Microbial Technology Institute, Shandong University, Qingdao, Shandong, China, 266237,

^2^School of Pharmaceutical Sciences, Shandong University, Jinan, Shandong, China, 250012

*Correspondence authors: Jinfeng Ni, jinfgni@sdu.edu.cn; Yaoyao Li, liyaoyao@sdu.edu.cn

**
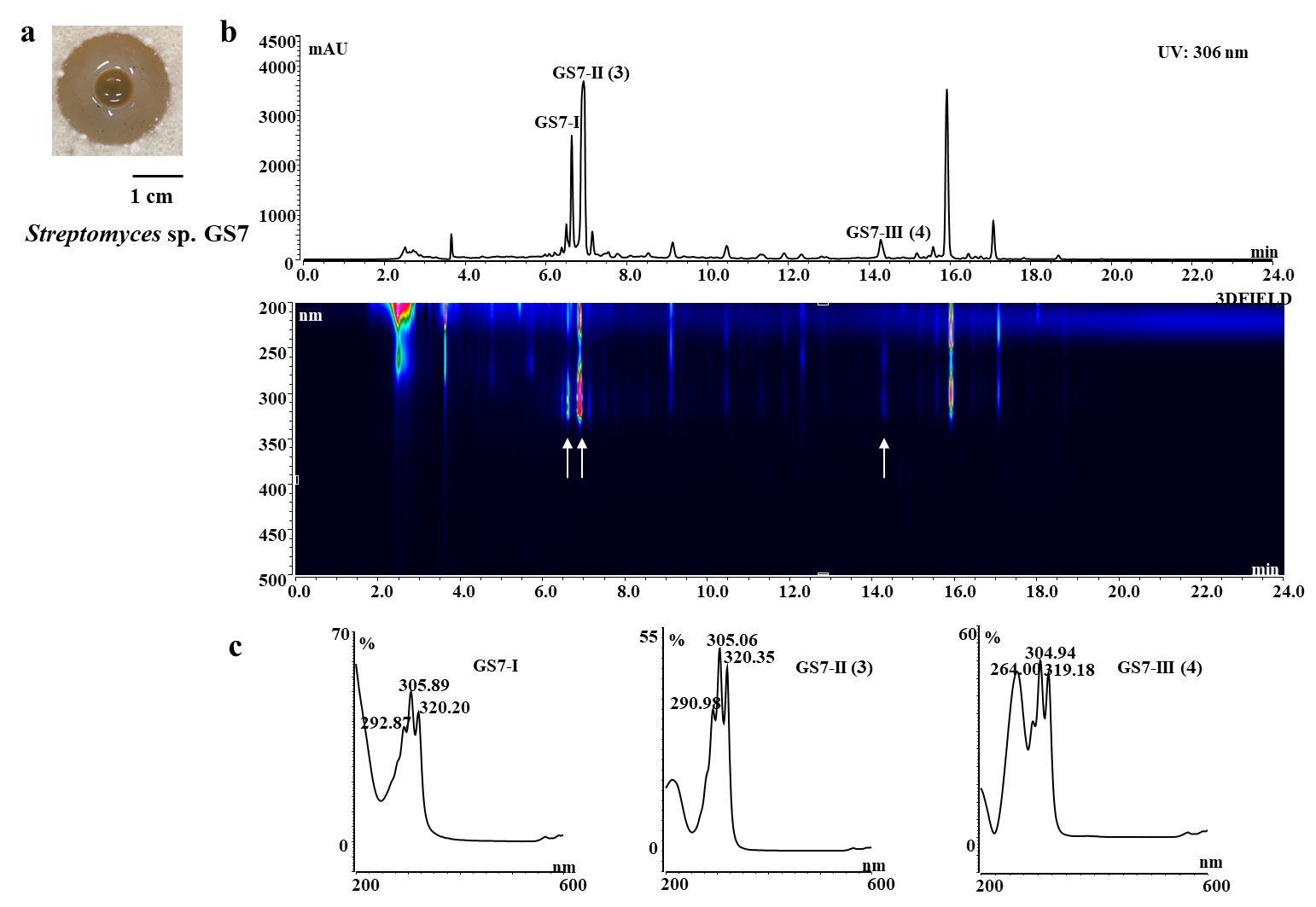
**

**Figure S1. Antifungal activity and HPLC analysis of the crude extract from *Streptomyces* sp. GS7.** (**a**) Antifungal activity of crude extract from *Streptomyces* sp. GS7 against entomopathogenic fungi (*M. anisopliae*) using the agar diffusion method. Bar: 1 cm. (**b**) HPLC analysis of the metabolites from *Streptomyces* sp. GS7. The arrows indicate peaks of three predicted polyenes GS7-I, GS7-II (**3**) and GS7-III (**4**) produced by *Streptomyces* sp. GS7. (**c**) UV/Vis spectra of GS7-I, GS7-II (**3**) and GS7-III (**4**).

**
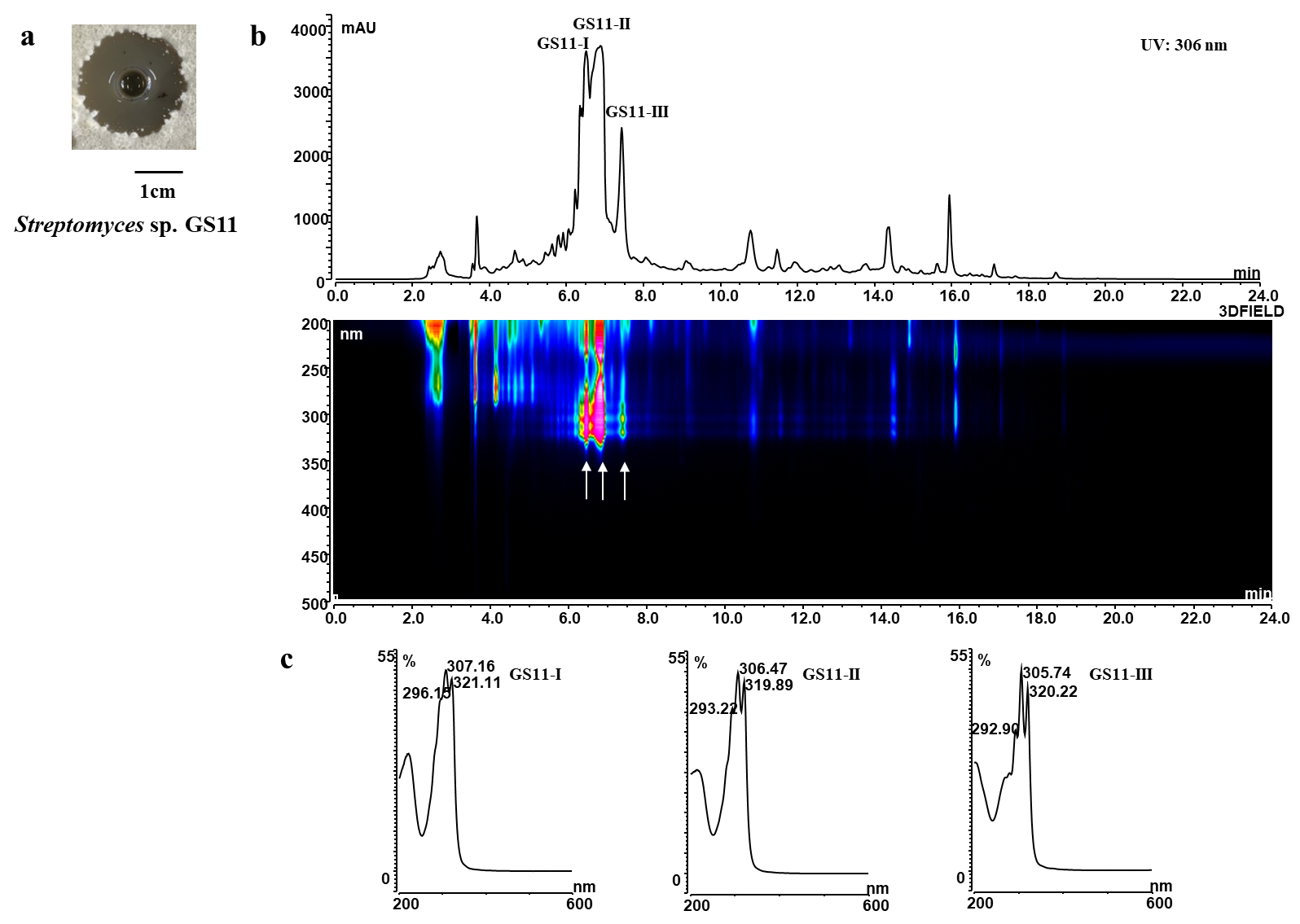
**

**Figure S2. Antifungal activity and HPLC analysis of the crude extract from *Streptomyces* sp. GS11.** (a) Antifungal activity of crude extract from *Streptomyces* sp. GS11 against entomopathogenic fungi (*M. anisopliae*) using the agar diffusion method. Bar: 1 cm. (**b**) HPLC analysis of the metabolites from *Streptomyces* sp. GS11. The arrows indicate peaks of three predicted polyenes GS11-I, GS11-II and GS11-III produced by *Streptomyces* sp. GS11. (**c**) UV/Vis spectra of GS11-I, GS11-II and GS11-III.

**
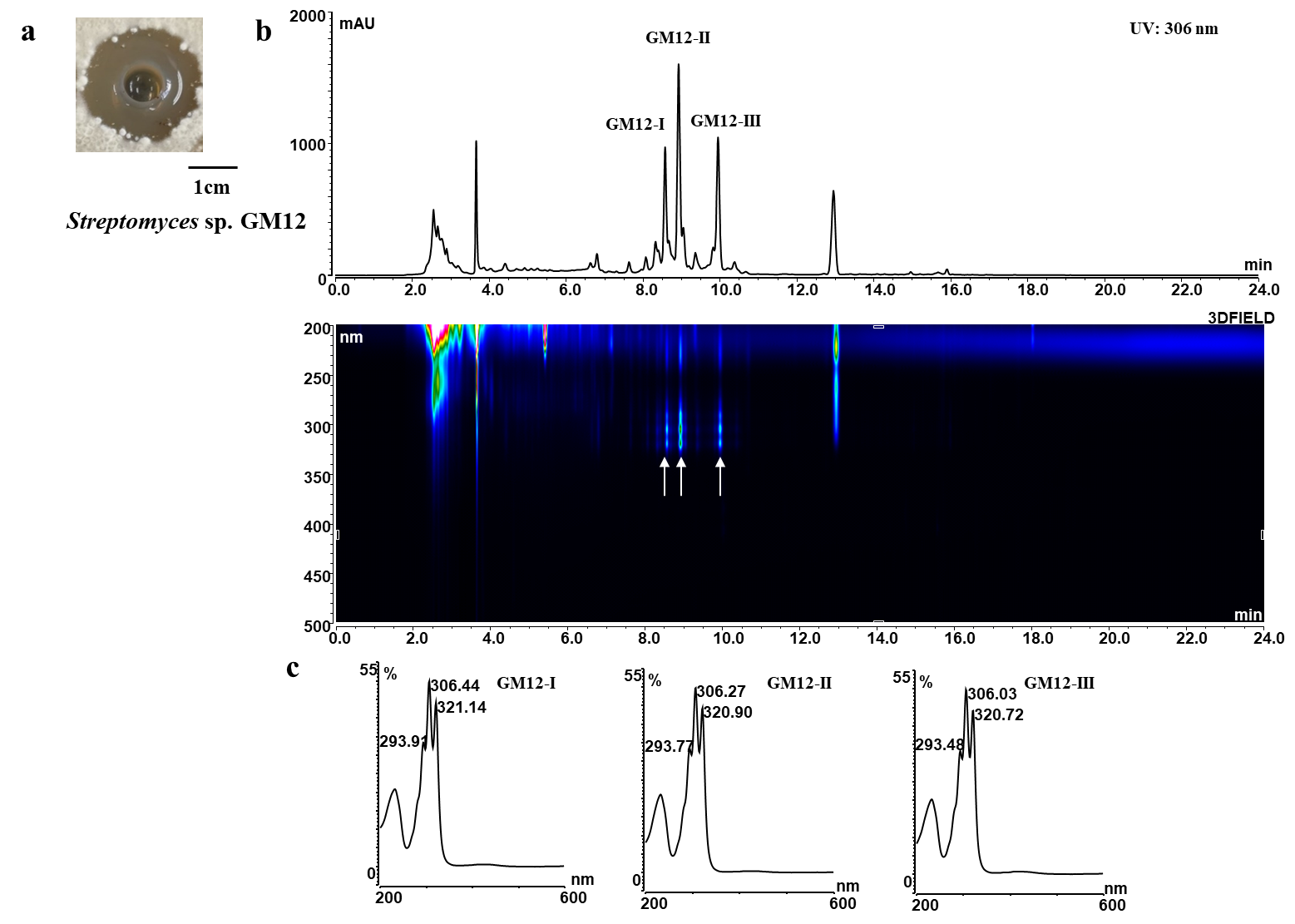
**

**Fig. S3. Antifungal activity and HPLC analysis of the crude extract from *Streptomyces* sp. GM12.** (**a**) Antifungal activity of crude extract from *Streptomyces* sp. GM12 against entomopathogenic fungi (*M. anisopliae*) using the agar diffusion method. Bar: 1 cm. (**b**) HPLC analysis of the metabolites from *Streptomyces* sp. GM12. The arrows indicate peaks of three predicted polyenes GM12-I, GM12-II and GM12-III produced by *Streptomyces* sp. GM12. (**c**) UV/Vis spectra of GM12-I, GM12-II and GM12-III.

**
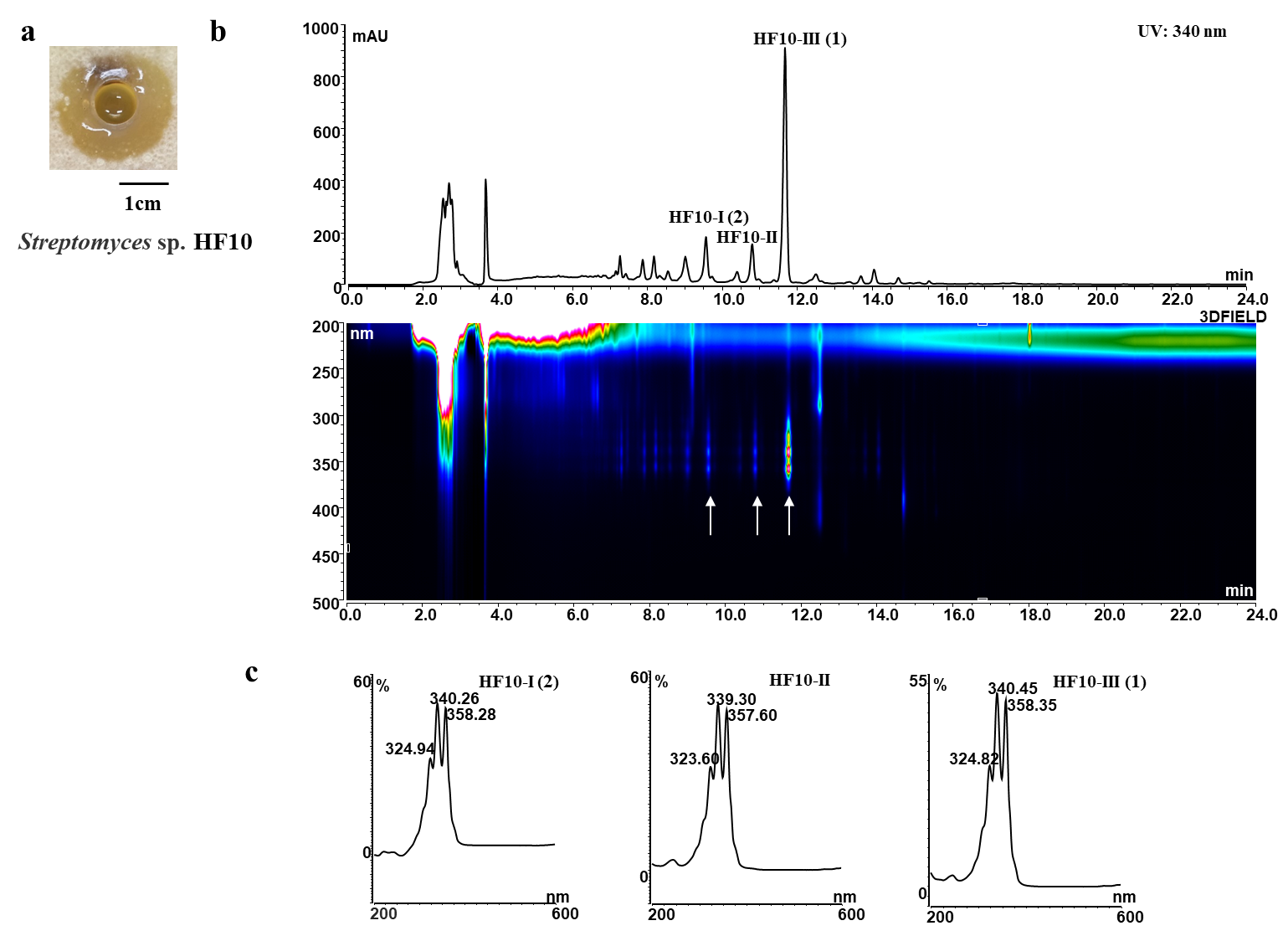
**

**Fig. S4 Antifungal activity and HPLC analysis of the crude extract from *Streptomyces* sp. HF10.** (**a**) Antifungal activity of crude extract from *Streptomyces* sp. HF10 against entomopathogenic fungi (*M. anisopliae*) using the agar diffusion method. Bar: 1 cm. (**b**) HPLC analysis of the metabolites from *Streptomyces* sp. HF10. The arrows indicate peaks of three predicted polyenes HF10-I (**2**), HF10-II and HF10-III (**1**) produced by *Streptomyces* sp. HF10. (**c**) UV/Vis spectra of HF10-I, HF10-II and HF10-III.

**
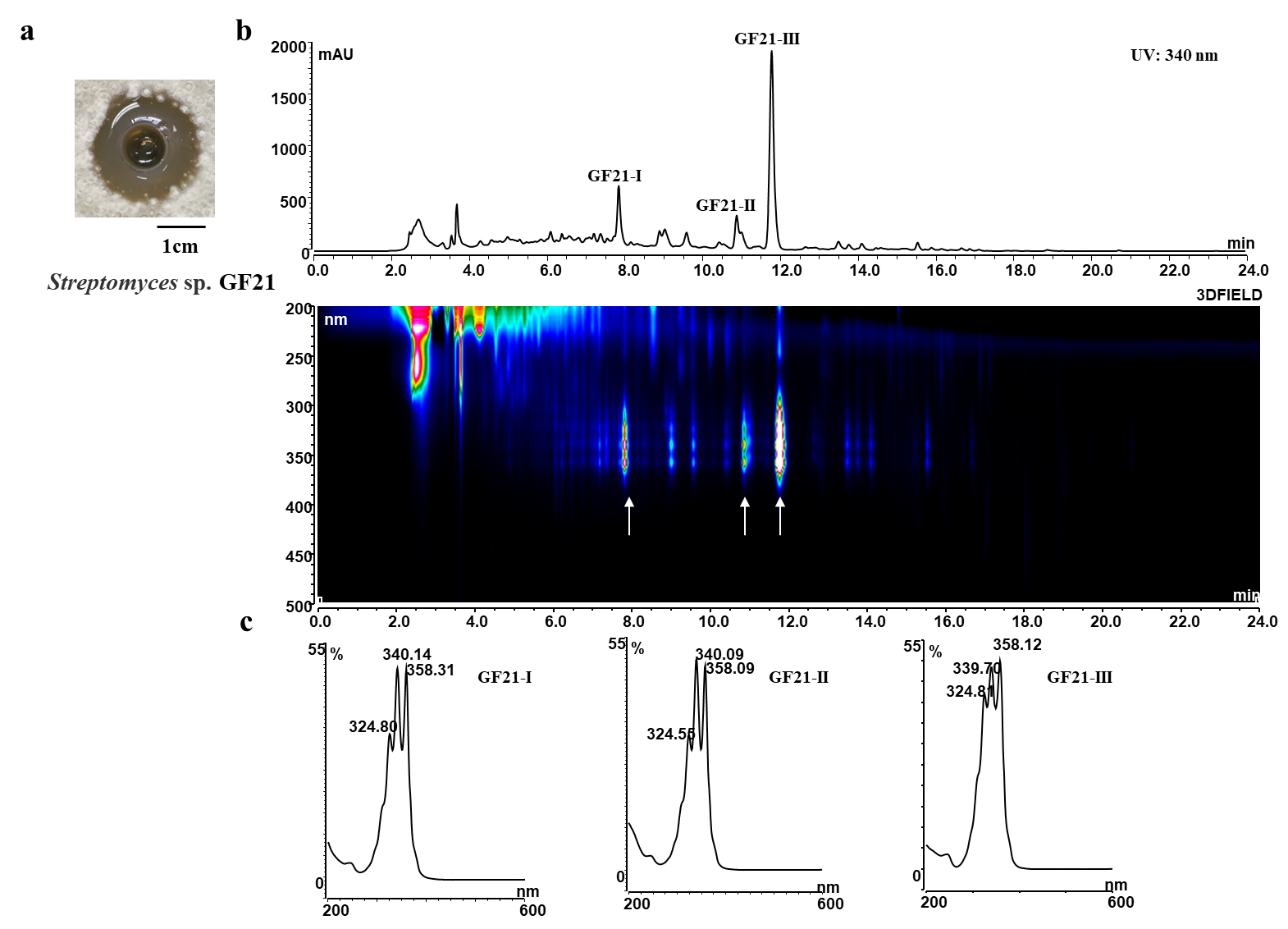
**

**Fig. S5 Antifungal activity and HPLC analysis of the crude extract from *Streptomyces* sp. GF21.** (**a**) Antifungal activity of crude extract from *Streptomyces* sp. GF21 against entomopathogenic fungi (*M. anisopliae*) using the agar diffusion method. Bar: 1 cm. (**b**) HPLC analysis of the metabolites from *Streptomyces* sp. GF21. The arrows indicate peaks of three predicted polyenes GF21-I, GF21-II and GF21-III produced by *Streptomyces* sp. GF21. (**c**) UV/Vis spectra of GF21-I, GF21-II and GF21-III.

**
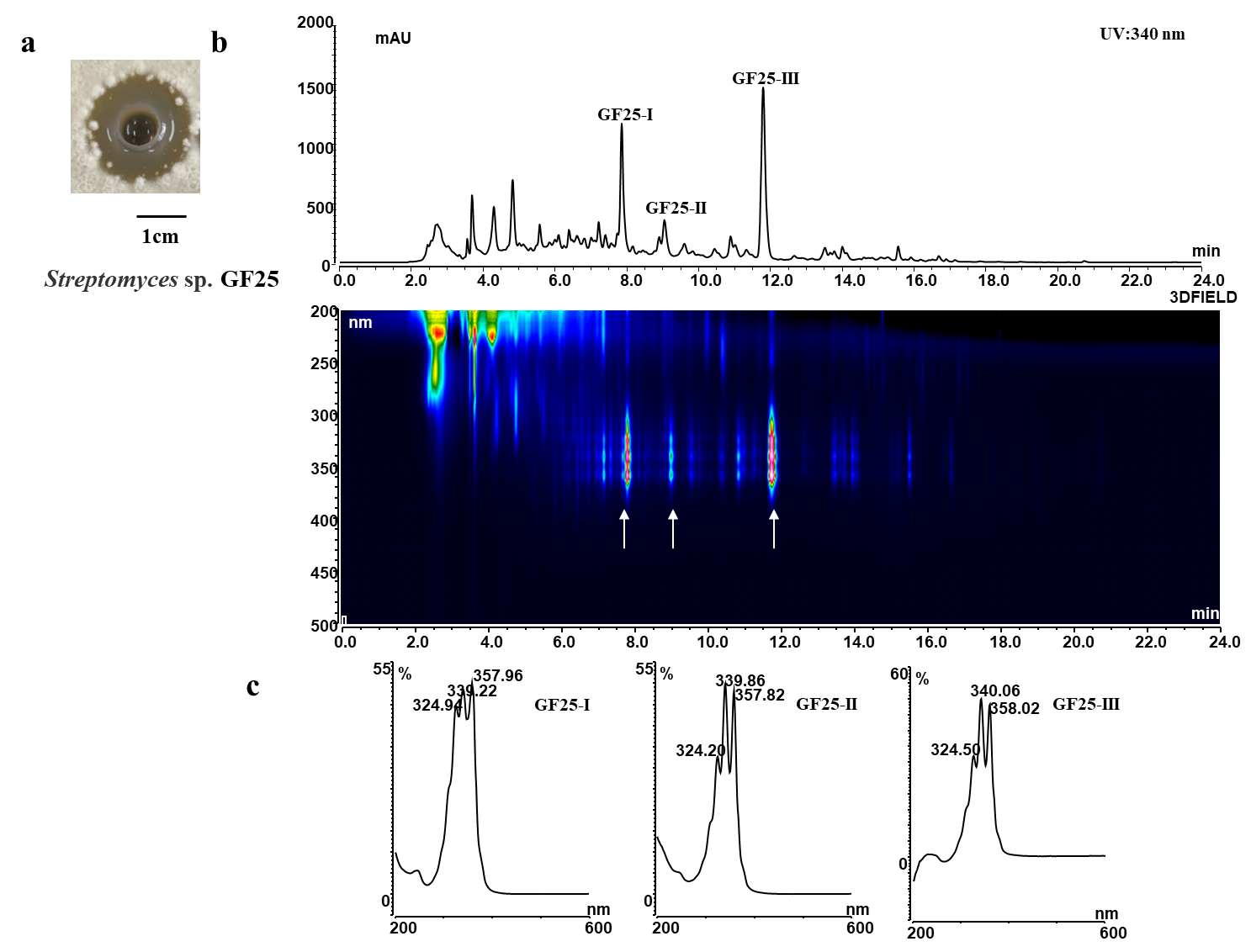
**

**Fig. S6 Antifungal activity and HPLC analysis of the crude extract from *Streptomyces* sp. GF25.** (**a**) Antifungal activity of crude extract from *Streptomyces* sp. GF25 against entomopathogenic fungi (*M. anisopliae*) using the agar diffusion method. Bar: 1 cm. (**b**) HPLC analysis of the metabolites from *Streptomyces* sp. GF25. The arrows indicate peaks of three predicted polyenes GF25-I, GF25-II and GF25-III produced by *Streptomyces* sp. GF25. (**c**) UV/Vis spectra of GF25-I, GF25-II and GF25-III.

**
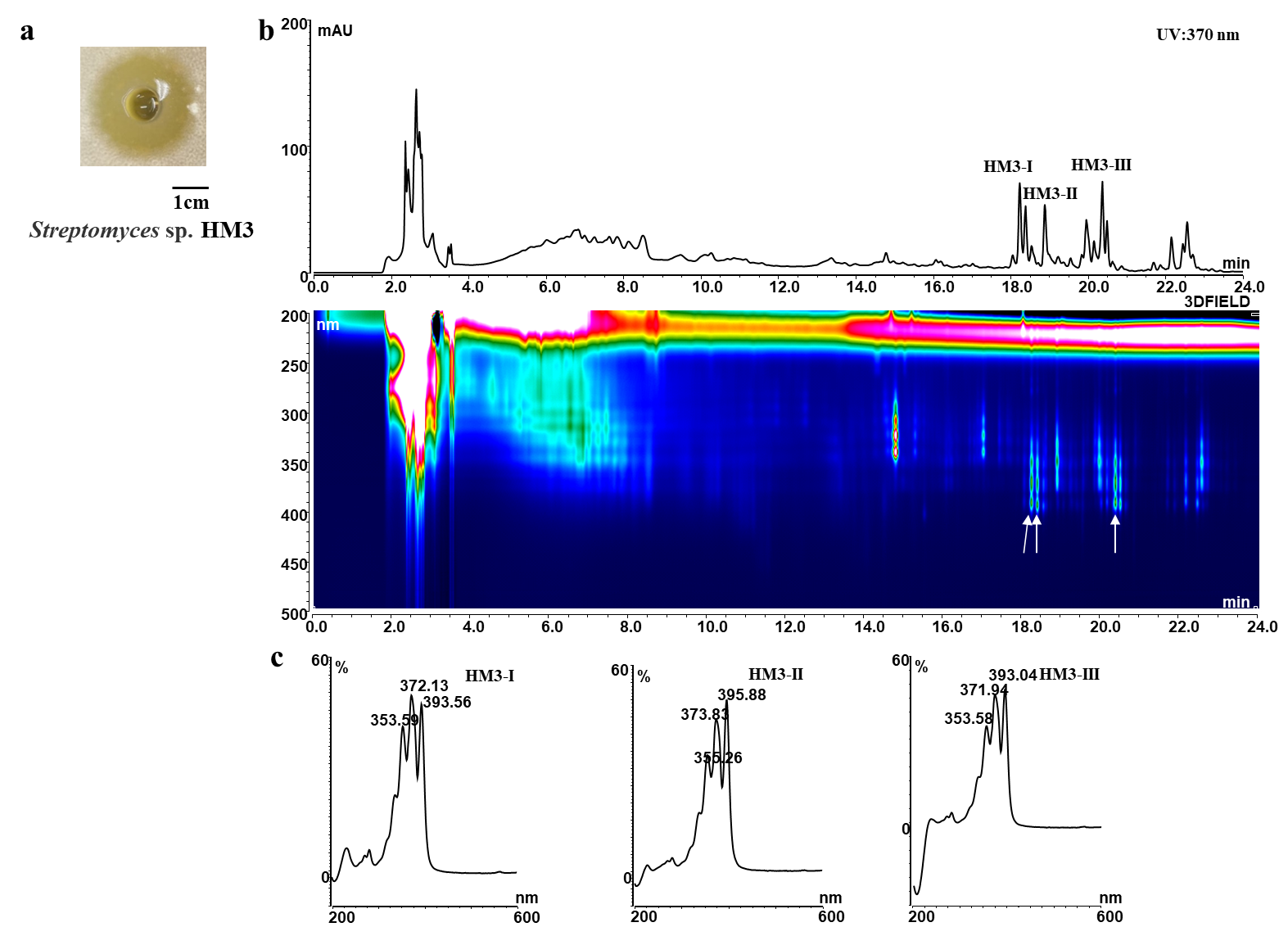
**

**Fig. S7 Antifungal activity and HPLC analysis of the crude extract from *Streptomyces* sp. HM3.** (**a**) Antifungal activity of crude extract from *Streptomyces* sp. HM3 against entomopathogenic fungi (*M. anisopliae*) using the agar diffusion method. Bar: 1 cm. (**b**) HPLC analysis of the metabolites from *Streptomyces* sp. HM3. The arrows indicate peaks of three predicted polyenes HM3-I, HM3-II and HM3-III produced by *Streptomyces* sp. HM3. (**c**) UV/Vis spectra of HM3-I, HM3-II and HM3-III.

**
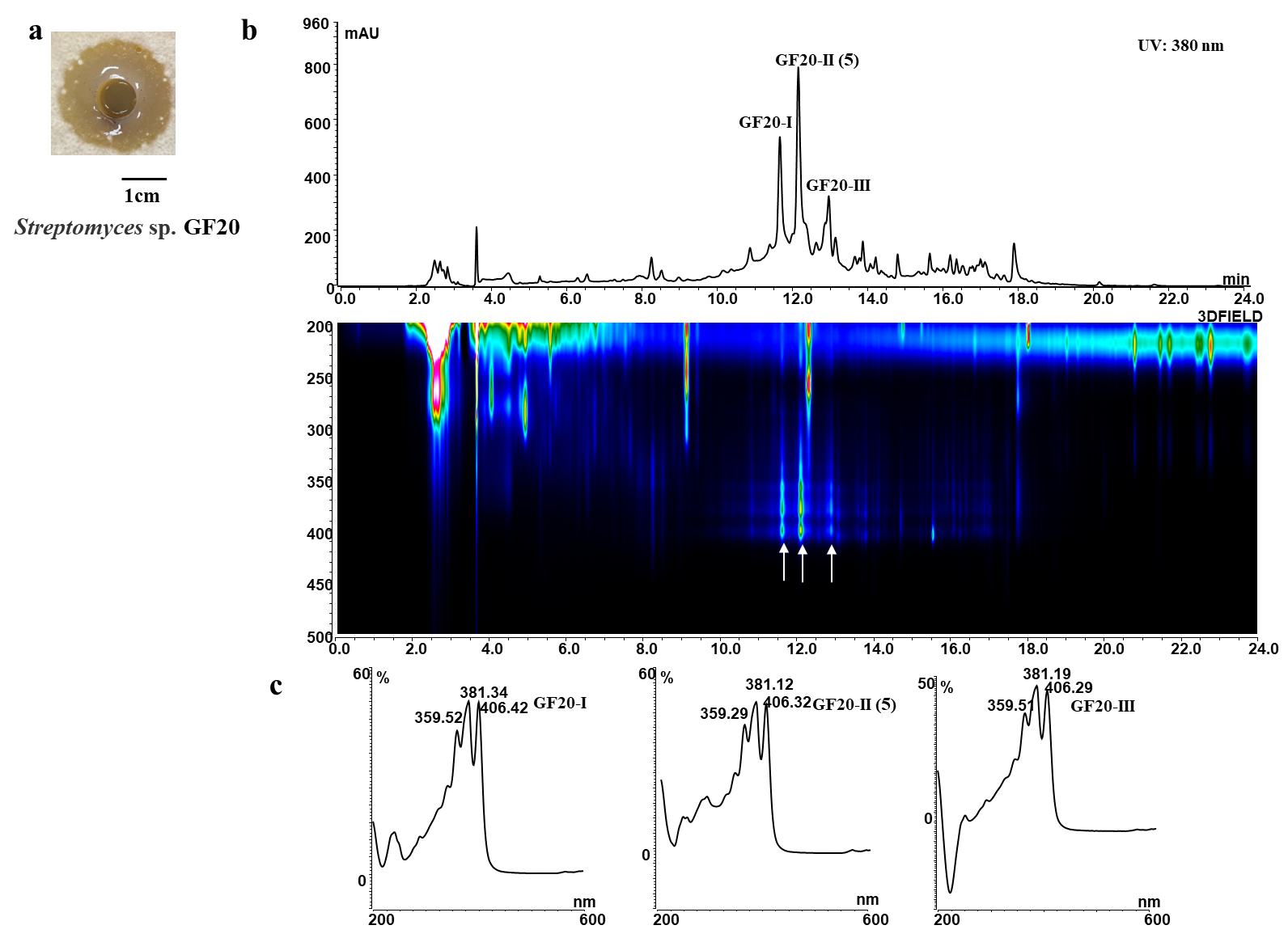
**

**Fig. S8 Antifungal activity and HPLC analysis of the crude extract from *Streptomyces* sp. GF20.** (**a**) Antifungal activity of crude extract from *Streptomyces* sp. GF20 against entomopathogenic fungi (*M. anisopliae*) using the agar diffusion method. Bar: 1 cm. (**b**) HPLC analysis of the metabolites from *Streptomyces* sp. GF20. The arrows indicate peaks of three predicted polyenes GF20-I, GF20-II (**5**) and GF20-III produced by *Streptomyces* sp. GF20. (**c**) UV/Vis spectra of GF20-I, GF20-II (**5**) and GF20-III.

**
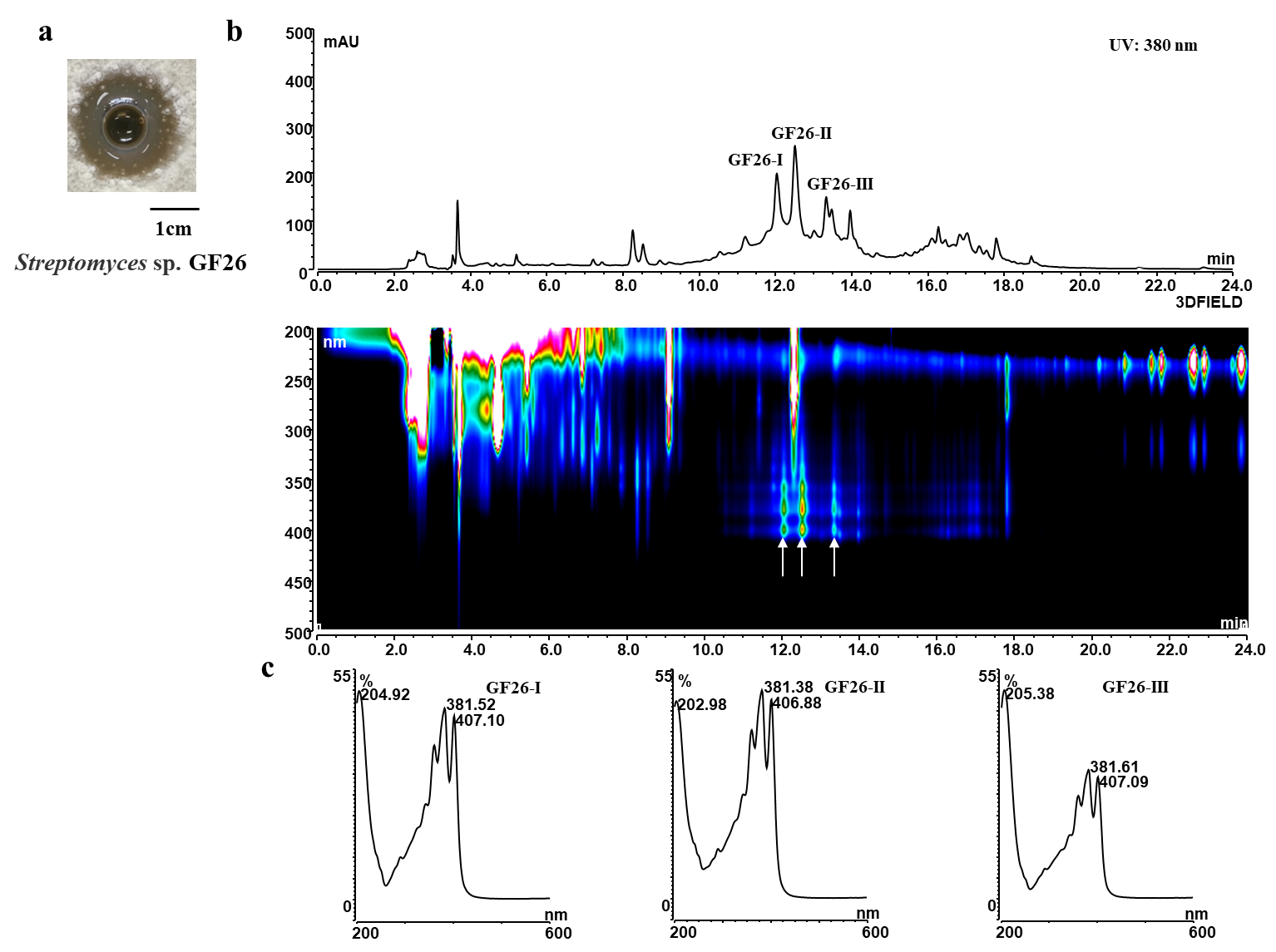
**

**Fig. S9 Antifungal activity and HPLC analysis of the crude extract from *Streptomyces* sp. GF26.** (**a**) Antifungal activity of crude extract from *Streptomyces* sp. GF26 against entomopathogenic fungi (*M. anisopliae*) using the agar diffusion method. Bar: 1 cm. (**b**) HPLC analysis of the metabolites from *Streptomyces* sp. GF26. The arrows indicate peaks of three predicted polyenes GF26-I, GF26-II (**5**) and GF26-III produced by *Streptomyces* sp. GF26. (**c**) UV/Vis spectra of GF26-I, GF26-II (**5**) and GF26-III.

**
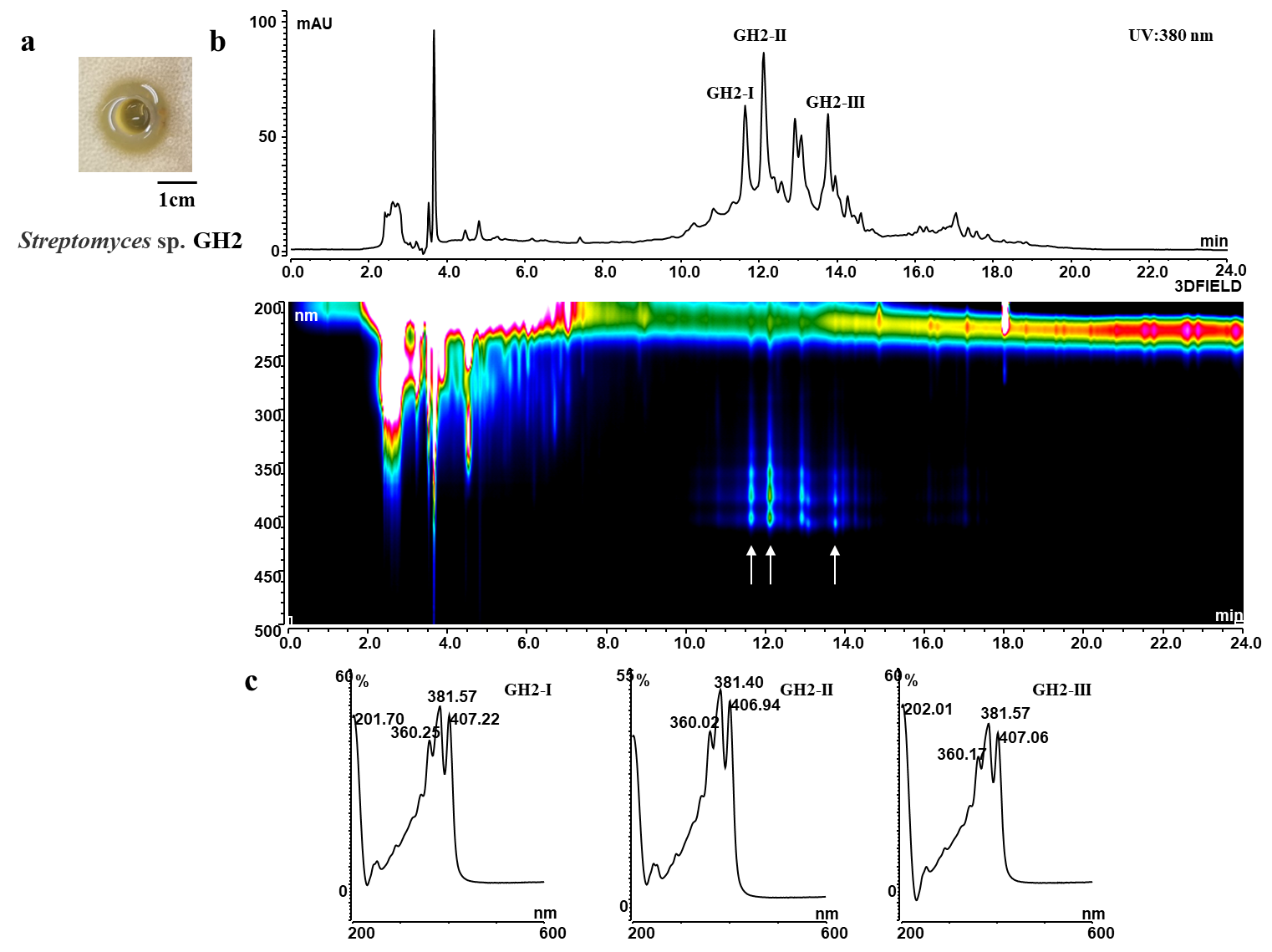
**

**Fig. S10 Antifungal activity and HPLC analysis of the crude extract from *Streptomyces* sp. GH2.** (**a**) Antifungal activity of crude extract from *Streptomyces* sp. GH2 against entomopathogenic fungi (*M. anisopliae*) using the agar diffusion method. Bar: 1 cm. (**b**) HPLC analysis of the metabolites from *Streptomyces* sp. GH2. The arrows indicate peaks of three predicted polyenes GH2-I, GH2-II (**5**) and GH2-III produced by *Streptomyces* sp. GH2. (**c**) UV/Vis spectra of GH2-I, GH2-II (**5**) and GH2-III.

**Figure S11.** HR-ESIMS spectrum of compound **1**.

**Figure S12.** HR-ESIMS spectrum of compound **2**.

**Figure S13.** ^1^H NMR spectrum of compound **1** in DMSO-*d*_6_.

**Figure S14.** ^13^C NMR spectrum of compound **1** in DMSO-*d*_6_.

**Figure S15.** The HSQC spectrum of compound **1** in DMSO-d6.

**Figure S16.** The HMBC spectrum of compound **1** in DMSO-d6.

**Figure S17**. The ^1^H-^1^H COSY spectrum of compound **1** in DMSO-*d*_6_.

**Figure S18**. The ROESY spectrum of compound **1** in DMSO-*d*_6_.

**Figure S19**. ^1^H NMR spectrum of compound **2** in DMSO-*d*_6_.

**Figure S20**. ^13^C NMR spectrum of compound **2** in DMSO-*d*_6_.
