# Supplementary material for: Polyene-Producing Streptomyces spp. From the Fungus-Growing Termite Macrotermes barneyi Exhibit High Inhibitory Activity Against the Antagonistic Fungus Xylaria
Source: Front Microbiol. 2021 Apr 1;12:649962. doi: 10.3389/fmicb.2021.649962 (PMC8047067; doi:10.3389/fmicb.2021.649962)
Supplement: Supplementary file 3 [file Table_2.docx]

**Supplementary table legends**

**Table S1** List of all actinobacterial strains isolated from *Macrotermes barneyi*.

**Table S2** Summary of strains which could represent new species (16S rRNA sequence similarity <98.65%).

**Table S3** Comparison of pentamycin biosynthetic gene cluster in *Streptomyces* sp. HF10 and *Streptomyces* sp. 816.

**Table S4** ^1^H and ^13^C NMR data for pentamycin (**1**) and 1'14-dihydroxyisochainin (**2**) in DMSO-*d_6_* (At 600 and 125 MHz, *δ* in ppm, *J* in Hz).

**Table S5** Comparison of putative natamycin biosynthetic gene cluster in *Streptomyces* sp. GS7 and *S. natalensis* ATCC 27448.

**Table S6** Comparison of putative candicidin biosynthetic gene cluster in *Streptomyces* sp. GF20 and *Streptomyces* sp. FR-008.

**Table S7** The primers used in the study.

**Table S2.** Summary of strains which are potentially new species (16S rRNA sequence similarity <98.65%).

| Strains | Source | Termite caste | Media | Inhibitor | Site of the gut | The closest species in the Genbank | Similarity (%) |
| --- | --- | --- | --- | --- | --- | --- | --- |
| HF5 | Hunan | Worker | Gause's No 1 | - | Foregut | *S. tacrolimicus* | 97.98 |
| HF17 | Hunan | Worker | Gause's No 1 | - | Foregut | *K. phosalacinea* | 98.58 |
| GF5 | Guangdong | Worker | M4 | - | Foregut | *S. bryophytorum* | 98.45 |
| GF6 | Guangdong | Worker | M4 | - | Foregut | *S. cocklensis* | 98.56 |
| GF15 | Guangdong | Worker | Gause's No 1 | - | Foregut | *S. bryophytorum* | 98.59 |
| GF18 | Guangdong | Worker | Gause's No 1 | - | Foregut | *S. cocklensis* | 98.28 |
| GM8 | Guangdong | Worker | Gause's No 1 | + | Midgut | *A. acidiphila* | 96.21 |

Note: +: Medium with inhibitor (50 μg/ml potassium dichromate); -: Medium without inhibitor.

**Table S3.** Comparison of pentamycin biosynthetic gene cluster in *Streptomyces* sp. HF10 and *Streptomyces* sp. 816.

| Proposed function | Pentamycin  (*Streptomyces* sp. HF10) | | Pentamycin  (*Streptomyces* sp. 816) | | Identity (%) |
| --- | --- | --- | --- | --- | --- |
|  | Gene （ID） | Aa No. | Gene (ID) | Aa No. |  |
| Thioesterase | *ptnH* (HF10_0321) | 255 | *ptnH (DV517_01290)* | 255 | 89.8 |
| PAS-LuxR_regulator | *ptnF* (HF10_0322) | 192 | *ptnF (DV517_01310)* | 192 | 93.2 |
| SARP-LAL regulator | *ptnR* (HF10_0324) | 1172 | *ptnR (DV517_01320)* | 1188 | 88.6 |
| Ferredoxin | *ptnE* (HF10_0325) | 80 | *ptnE (DV517_01330)* | 80 | 85.0 |
| Cytochrome P450 (hydroxylation of C-26) | *ptnD* (HF10_0326) | 404 | *ptnD (DV517_01340)* | 404 | 98.0 |
| Cytochrome P450 (hydroxylation of C-1’) | *ptnC* (HF10_0327) | 399 | *ptnC (DV517_01350)* | 399 | 98.0 |
| Crotonyl-CoA_reductase | *ptnB* (HF10_0328) | 419 | *ptnB (DV517_01360)* | 418 | 97.7 |
| PKS | *ptnA5* (HF10_0329) | 3365 | *ptnA5 (DV517_01370)* | 3353 | 92.0 |
| PKS | *ptnA4* (HF10_0330) | 6207 | *ptnA4 (DV517_01380)* | 6153 | 90.6 |
| PKS | *ptnA3* (HF10_0331) | 1816 | *ptnA3 (DV517_01390)* | 1823 | 89.6 |
| PKS | *ptnA2* (HF10_0332) | 3615 | *ptnA2 (DV517_01400)* | 3613 | 90.6 |
| PKS | *ptnA1* (HF10_0333) | 7820 | *ptnA1 (DV517_01410)* | 7840 | 89.0 |
| Cytochrome P450 (hydroxylation of C-14) | *ptnI* (HF10_0334) | 366 | *ptnJ*(DV517_01420) | 405 | 96.0 |
| Ferredoxin | *ptnJ* (HF10_0335) | 64 | *ptnI*(DV517_01430) | 64 | 98.0 |

**Table S4.** ^1^H and ^13^C NMR data for Pentamycin (**1**) and 1'14-dihydroxyisochainin (**2**) in DMSO-*d_6_* (At 600 and 125 MHz, *δ* in ppm, *J* in Hz).

| No. | Pentamycin (**1**) | | 1'14-dihydroxychainin (**2**) | |
| --- | --- | --- | --- | --- |
|  | ^1^H | ^13^C | ^1^H | ^13^C |
| 1 | - | 171.5s | - | 171.5s |
| 2 | 2.45 (dd, 8.5, 7.1) | 59.2d | 2.44 (t, 7.9) | 59.3d |
| 3 | 3.99-3.93 (m) | 70.8d | 3.99-3.93 (m) | 70.9d |
| 4 | 1.38-1.31 (m) | 40.6t | 1.38-1.31 (m) | 40.5t |
| 5 | 3.89-3.85 (m) | 70.7d | 3.89-3.85 (m) | 70.8d |
| 6 | 1.30-1.25 (m) | 44.3t | 1.30-1.25 (m) | 44.3t |
| 7 | 3.88-3.84 (m) | 70.5d | 3.88-3.84 (m) | 70.5d |
| 8 | 1.39-1.34 (m)  1.30-1.24 (m) | 44.5t | 1.39-1.34 (m)  1.30-1.24 (m) | 44.5t |
| 9 | 3.86-3.81 (m) | 71.6d | 3.86-3.81 (m) | 71.6d |
| 10 | 1.37-1.32 (m)  1.26-1.23 (m) | 43.3t | 1.37-1.32 (m)  1.26-1.23 (m) | 43.3t |
| 11 | 3.77-3.74 (m) | 69.7d | 3.77-3.74 (m) | 69.7d |
| 12 | 1.58-1.53 (m)  1.36-1.31 (m) | 39.1t | 1.58-1.53 (m)  1.36-1.31 (m) | 39.1t |
| 13 | 3.10 (d, 10.6) | 68.8d | 3.10 (d, 10.7) | 68.8d |
| 14 | 3.46 (d, 9.0) | 77.0d | 3.46 (d, 9.0) | 77.0d |
| 15 | 3.67-3.64 (m) | 78.6d | 3.67-3.64 (m) | 78.6d |
| 16 | - | 139.3s | - | 139.3s |
| 17 | 5.93 (br d, 11.3) | 127.6d | 5.92 (br d, 11.3) | 127.6d |
| 18 | 6.45 (14.4, 11.5) | 128.4d | 6.45 (14.5, 11.2) | 128.4d |
| 19 | 6.25-6.21 (m) | 133.5d | 6.25-6.21 (m) | 133.6d |
| 20 | 6.34-6.31 (m) | 132.8d | 6.34-6.31 (m) | 132.8d |
| 21 | 6.23-6.19 (m) | 131.7d | 6.23-6.19 (m) | 131.7d |
| 22 | 6.34-6.29 (m) | 129.5d | 6.34-6.29 (m) | 129.6d |
| 23 | 6.38 (dd, 14.2, 10.7) | 133.8d | 6.38 (dd, 13.9, 10.9) | 133.8d |
| 24 | 6.33-6.30 (m) | 133.7d | 6.33-6.30 (m) | 133.7d |
| 25 | 6.03 (dd, 14.6, 4.4) | 135.5d | 6.03 (dd, 14.6, 4.4) | 135.5d |
| 26 | 3.97-3.92 (m) | 71.6d | 3.97-3.92 (m) | 71.6d |
| 27 | 4.62 (quint, 6.4) | 73.6d | 4.62 (quint, 6.8) | 73.7d |
| 28 | 1.17 (d, 6.3) | 18.3q | 1.17 (d, 6.4) | 18.2q |
| 29 | 1.67 (s) | 12.1q | 1.66 (s) | 12.1q |
| 1´ | 3.65-3.62 (m) | 70.0d | 3.65-3.62 (m) | 69.8d |
| 2´ | 1.35-1.30 (m)  1.25-1.20 (m) | 34.6t |  | 36.8t |
| 3´ | 1.42-1.38 (m)  1.24-1.21 (m) | 25.1t |  | 18.7t |
| 4´ | 1.23-1.18 (m)  1.18-1.14 (m) | 31.7t | 0.83 (t, 6.9) | 14.3q |
| 5´ | 1.25-1.19 (m) | 22.6t |  |  |
| 6´ | 0.85 (t, 6.8) | 14.4q |  |  |

**Table S5**. Comparison of putative natamycin biosynthetic gene cluster in *Streptomyces* sp. GS7 and *S. natalensis* ATCC 27448.

| Proposed function | Natamycin  (*Streptomyces* sp. GS7) | | Natamycin  (*S. natalensis* ATCC 27448) | | Identity  (%) |
| --- | --- | --- | --- | --- | --- |
|  | Gene (ID) | Aa No. | Gene (ID) | Aa No. |  |
| Major facilitator transporter | *-* | - | *PimH (CAC20933)* | 432 | - |
| DegT/DnrJ/EryC1/StrS aminotransferase | *PimC (GS7_004616)* | *352* | *PimC (CAC20927)* | 352 | 94.0 |
| Cytochrome P450 | *PimG (GS7_004617)* | 413 | *PimG* (CAC20928*)* | 398 | 84.8 |
| Ferredoxin | *PimF (GS7_004618)* | 63 | *PimF (CAC20929)* | 63 | 82.6 |
| PKS | *PimS0 (GS7_004619)* | 1743 | *PimS0 (CAC20930)* | 1766 | 82.3 |
| PKS | *PimS1 (GS7_004621)* | 6795 | *PimS1(CAC20931)* | 6805 | 83.1 |
| Cytochrome P450 | *PimD (GS7_004622)* | 397 | *PimD (CAC20932)* | 397 | 88.4 |
| Glycosyltransferase | *PimK (GS7_004623)* | 458 | *PimK (CAC20918)* | 458 | 88.7 |
| PKS | *PimS4(GS7_004624)* | 2033 | *PimS4 (CAC20919)* | 2024 | 81.4 |
| PKS | *PimS3(GS7_004625)* | 1817 | *PimS3 (CAC20920)* | 1808 | 82.4 |
| PKS | *PimS2(GS7_004626)* | 9526 | *PimS2 (CAC20921)* | 9507 | 86.4 |
| LuxR transcriptional regulator | *PimR1(GS7_004628)* | 926 | *-* | - | - |
| LuxR transcriptional regulator | *PimR2(GS7_004629)* | 937 | *-* | - | - |
| LuxR transcriptional regulator | *PimR3(GS7_004630)* | 962 | *-* | - | - |
| Thioesterase | *PimI (GS7_004631)* | 255 | *PimI (CAC20922)* | 255 | 88.1 |
| NAD-dependent epimerase/dehydratase | *PimJ (GS7_004632)* | 344 | *PimJ (CAC20923)* | 343 | 94.8 |
| ABC transporter related protein | *PimA(GS7_004633)* | 602 | *PimA (CAC20924)* | 602 | 85.4 |
| ABC transporter related protein | *PimB (GS7_004634)* | 602 | *PimB (CAC20925)* | 626 | 86.9 |
| SARP transcriptional regulator | *-* | - | *PimR (CAC20918)* | 1097 | - |
| Cholesterol oxidase | *PimE* | *616* | *PimE (CAC20926)* | 549 | 91.6 |

**Table S6.** Comparison of putative candicidin biosynthetic gene cluster in *Streptomyces* sp. GF20 and *Streptomyces* sp. FR-008.

| Proposed function | Candicidin  (*Streptomyces* sp. GF20) | | Candicidin  (*Streptomyces* sp. FR-008) | | Identity  (%) |
| --- | --- | --- | --- | --- | --- |
|  | Gene (ID) | Aa No. | Gene (ID) | Aa No. |  |
| FAD-dependent monooxygenase | *fscO(GF20_ 000112)* | 405 | *fscO (AAQ82549)* | 458 | 99.8 |
| 4-amino-4-deoxychorismate lyase | *fscC (GF20_ 000113)* | 257 | *pabC (AAQ82550)* | 257 | 98.4 |
| LuxR transcriptional regulator | *fscRI (GF20_000114)* | 231 | *fscRI (AAQ82551)* | 222 | 100 |
| LuxR transcriptional regulator | *fscRII (GF20_000115)* | 942 | *fscRII (AAQ82552)* | 942 | 99.9 |
| LuxR transcriptional regulator | *fscRIII(GF20_000116)* | 1009 | *fscRIII (AAQ82553)* | 1014 | 99.3 |
| LuxR transcriptional regulator | *fscRIV (GF20_000117)* | 970 | *fscRIV (AAQ82554)* | 1005 | 99.4 |
| Glycosyltransferase | *fscMI (GF20_000118)* | 458 | *fscMI (AAQ82555)* | 501 | 99.6 |
| DegT/DnrJ/EryC1/StrS aminotransferase | *fscMII (GF20_000119)* | 352 | *fscMII (AAQ82556)* | 352 | 100 |
| Cytochrome P450 | *fscP (GF20_000120)* | 393 | *fscP (AAQ82557)* | 421 | 100 |
| Ferredoxin | *fscFE (GF20_000121)* | 64 | *fscFE (AAQ82558)* | 64 | 100 |
| Thioesterase | *fscTE (GF20_000122)* | 256 | *fscTE (AAQ82559)* | 285 | 99.6 |
| Isochorismate synthase | *fscAB (GF20_000123)* | 721 | *pabAB (AAQ82560)* | 723 | 98.9 |
| PKS | *fscA (GF20_000124)* | 1742 | *fscA (AAQ82561)* | 1743 | 98.9 |
| ABC transporter | *fscTI (GF20_000125)* | 335 | *fscTI (AAQ82562)* | 335 | 100 |
| ABC transporter | *fscTII (GF20_000126)* | 280 | *fscTII (AAQ82563)* | 239 | 100 |
| PKS | *fscC (GF20_000127)* | 10627 | *fscC (AAQ82564)* | 10625 | 98.5 |
| PKS | *fscB ( GF20_000128)* | 5540 | *fscB (AAQ82565)* | 5541 | 98.7 |
| PKS | *fscF (GF20_000129)* | 2049 | *fscF (AAQ82566)* | 2049 | 99.1 |
| PKS | *fscE (GF20_000130)* | 7771 | *fscE (AAQ82567)* | 7771 | 99.2 |
| PKS | *fscD (GF20_000131)* | 9545 | *fscD (AAQ82568)* | 9550 | 99.2 |
| NAD-dependent epimerase/dehydratase | *fscMIII (GF20_000132)* | 344 | *fscMIII (AAQ82569)* | 402 | 100 |

**Table S7.** The primers used in the study.

| Primers | Sequences (5′-3′) |
| --- | --- |
| dP4LF | gttgtaaaacgacggccagtgaattcCGCGCCTTCGCGGACTACAA |
| dP4LR | gtcgacggatccccggaatTCGGCGAGGAGGGTGACGAGCC |
| *aac(3)IV-*P4F | ggctcgtcaccctcctcgccgaATTCCGGGGATCCGTCGAC |
| *aac(3)IV-*P4R | cgcaagcaccgtcttgatgaccTGTAGGCTGGAGCTGCTTCGA |
| dP4RF | tcgaagcagctccagcctacaGGTCATCAAGACGGTGCTTGCG |
| dP4RR | gctatgaccatgattacgccaagcttTGGTCGGGGACGAGACGGAAC |
| S0vF | TATAGACATCGACATGCGTATCACCGTT |
| S0vR | TAATCAGCGCAAGCACCGTCTTGA |
| dP7-PstI-LF | TGACTGCAGCACGAGACCACGGCGAACAAC |
| dP7-XbaI-LR | GAATCTAGATGGGACCGAAGTCCAGGAACG |
| *aac(3)IV*-XbaI-F | TGGTCTAGAGGTTCATGTGCAGCTCCATCAG |
| *aac(3)IV*-XbaI-R | CGCTCTAGAATGAGCTCAGCCAATCGACTGG |
| dP7-XbaI-RF | CCATCTAGATTCACCGAGCTGGGCGTCAC |
| dP7-HindIII-RR | TCGAAGCTTGCGGACTGCTTTGCTGTGGG |
| AvF | TAATGTCTGCCAGCAGGAGATAGCGG |
| AvR | TTAAGGGGTGGTCGAAGATCAGGGTC |

The underlined indicates restriction enzyme sites.
